# Nitrogen-containing secondary metabolites from Meliaceae Family and their biological activity: a review

**DOI:** 10.1007/s13659-025-00531-w

**Published:** 2025-07-29

**Authors:** Ni Wayan Martiningsih, Siska Elisahbet Sinaga, Wahyu Safriansyah, Unang Supratman, Desi Harneti

**Affiliations:** 1 Department of Chemistry, Faculty of Mathematics and Natural Sciences, Universitas Padjadjaran, Jatinangor, 45363 Sumedang, West Java Indonesia; 2 Department of Chemistry, Faculty of Mathematics and Natural Sciences, Universitas Pendidikan Ganesha, Singaraja, 81116 Bali Indonesia; 3 Department of Food, Life, and Environmental Science, Faculty of Agriculture, Yamagata University, Tsuruoka, Yamagata 997-8555 Japan; 4 Central Laboratory, Universitas Padjadjaran, Jatinangor, 45363 Sumedang, West Java Indonesia

**Keywords:** Nitrogen-containing, Secondary metabolites, Meliaceae, Biological activity

## Abstract

The Meliaceae family, widely distributed in tropical and subtropical regions, has been traditionally used for medicinal purposes, particularly in treating infections and inflammatory diseases. The objective of this study is to provide a comprehensive account of the nitrogen-containing secondary metabolites and their biological activities that have been isolated from the Meliaceae family between the years 1979 and 2024. Studies on nitrogen-containing compounds of the Meliaceae family were collected and analyzed using data from SciFinder, PubMed, Google Scholar, Scopus and World Flora Online. Over the course of more than four decades, numerous studies have been conducted on a variety of plant parts, including twigs, stems, barks, roots, fruits, seeds, and leaves. These studies have identified approximately 326 compounds belonging to diverse chemical groups, such as alkaloids, limonoids, triterpenoids, and nitrogen-containing flavaglines being the largest group of natural products, comprising 118 compounds (36.2%). Several compounds have been evaluated for insecticidal, anti-inflammatory, molluscicide, antibacterial, antimalarial, tyrosinase inhibition, osteoclast differentiation inhibition, and α-glucosidase inhibition activity. The systematic classification and analysis of these compounds provide insights into their biosynthesis and bioactivities, paving the way for future drug development.

**Graphical Abstract:**

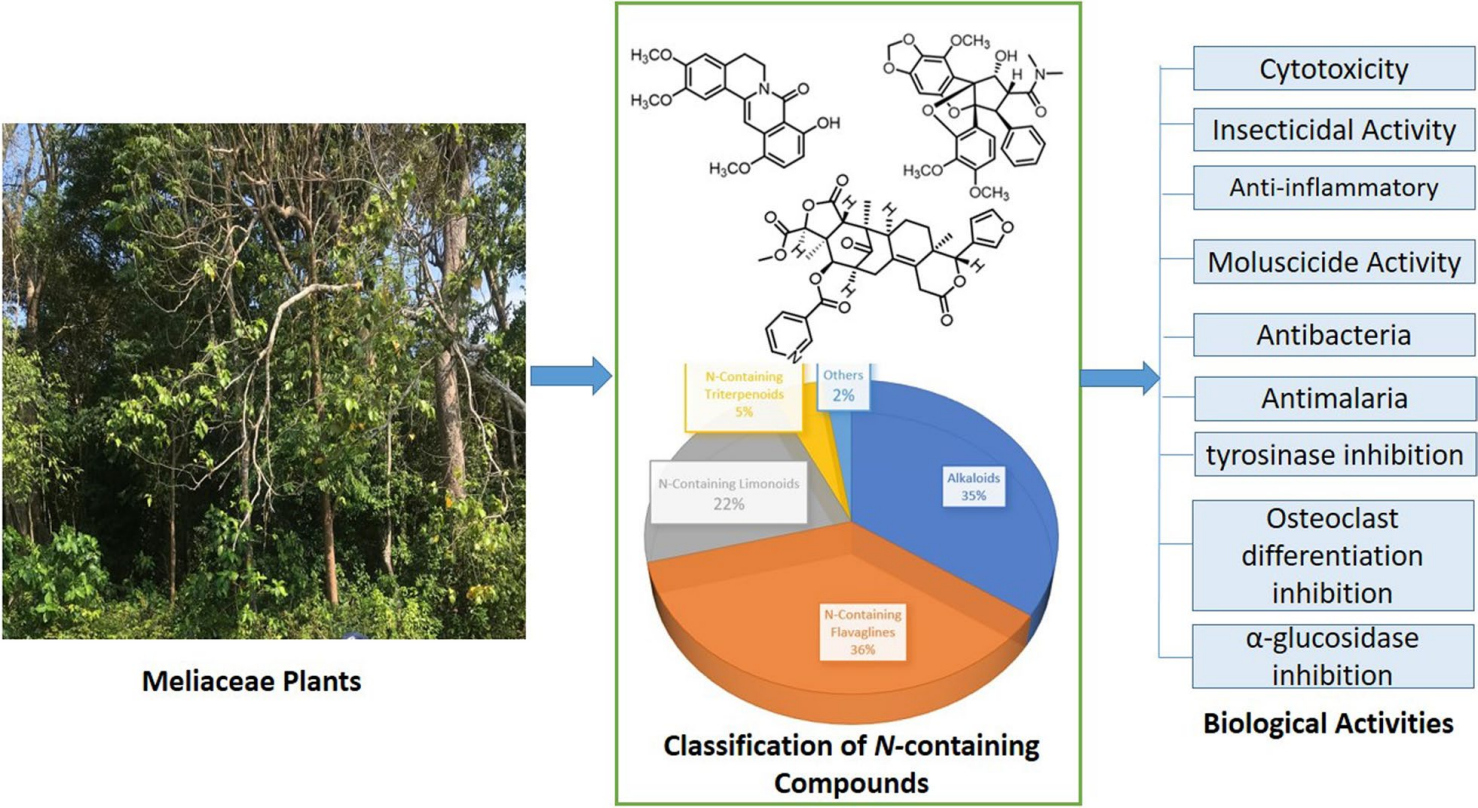

## Introduction

Natural compounds containing nitrogen, such as alkaloids, peptides, amino acids, nucleic acids, and sphingophospholipids, have been extracted from various organisms including plants, animals, fungi, insects, and marine life [1]. These compounds show a wide range of biological effects, including antitumor, antiviral, antibacterial, and immunosuppressive activity. Nitrogen-containing natural products have proved successful as drug leads and chemical probes for research. Some examples include vinblastine, vincristine, morphine, and quinine [2, 3].

The Meliaceae family, widely known for its diverse species like *Azadirachta indica* A. Juss. (neem) and *Aglaia odorata* Lour., has been used traditionally across cultures for its medicinal and insecticidal properties. Ancient herbal practices in regions like India, China, and Southeast Asia included remedies derived from Meliaceae for ailments such as infections, fevers, and inflammations. Over centuries, the use of these plants spread as explorers and trade routes carried knowledge and plant samples to new parts of the world. This family, commonly known as the mahogany family, consists of woody plants distributed across tropical and subtropical regions worldwide [4, 5]. Several decades of phytochemical research on the Meliaceae family has led to the identification of a range of secondary metabolites including triterpenoids [6–10], diterpenoids [11–13], sesquiterpenoids [14–16], steroids [17–19], limonoids [20–22], flavonoids [23, 24], lignans [25], flavaglines [26, 27], and alkaloids [28, 29].

The first phytochemical investigation of nitrogen-containing compounds from Meliaceae plant was conducted in 1979. This study led to the isolation of a chromone alkaloid, rohitukine from the leaves of *Dysoxylum acutangulum* Miq. and two bisamides, roxburghilin and odorinol, from the leaves of *Aglaia odorata* Lour. [30–32]. By the mid-twentieth century, advances in isolation techniques and bioassays allowed researchers to test these compounds’ effects against various pathogens and cancer cells. Early findings inspired more in-depth studies of Meliaceae compounds’ structures, mechanisms of action, and potential as pharmaceutical agents. The discovery of highly potent compounds, such as rohitukine and rocaglamide, marked a breakthrough, and their activities inspired further synthetic modifications to enhance efficacy. Flavopiridol, a semi-synthetic derivative of rohitukine, emerged as a promising anticancer agent undergoing clinical trials, exemplifying how traditional knowledge was transformed through modern science into potential therapies. Research conducted over four subsequent decades identified 5 classes of nitrogen-containing compounds. The current research continues this trajectory, assessing cytotoxic activities of various Meliaceae-derived alkaloids and flavaglines against a broad spectrum of cancer cell lines. By building on historical foundations and evolving research methodologies, these studies are crucial in uncovering the pharmacological potential of Meliaceae compounds. This historical perspective highlights the continuity of scientific inquiry and innovation rooted in ancient practices, showcasing the value of Meliaceae plants as a source of anticancer agents. Here we present the first comprehensive review of nitrogen-containing compounds isolated from plants in the Meliaceae family covering a total of 326 compounds. More importantly, these nitrogen-containing compounds, which have diverse structural characteristics, also demonstrate a wide range of pharmacological activities. These include cytotoxic [33–35], insecticidal [36–38], anti-inflammatory [26, 39, 40], molluscicide [41–43], antibacterial [44], and antimalarial agents [45]. Some nitrogen-containing compounds have formed the basis for the development of new drugs, and further research is ongoing to optimize their use in modern medicine.

## Material and methods

A systematic literature search was conducted using SciFinder, PubMed, Google Scholar, Scopus, and World Flora Online. Articles published between 1979 and 2024 were selected based on their relevance to nitrogen-containing compounds in Meliaceae. Inclusion criteria encompassed studies that reported structural elucidation, biological activity, and biosynthetic pathways of these metabolites. Duplicate reports and studies lacking detailed characterization were excluded.

## Plant distribution and habitats

Plants of the Meliaceae family are woody plants belonging to the mahogany group, classified under the order Sapindales and the class Angiospermae. The Meliaceae family is widely distributed across tropical regions in Asia, Africa, and the Americas. In addition, it has a notable presence outside the tropics in areas such as South Africa, New Zealand, central China, northern India, Nepal, Pakistan, and Australia [46].

The Meliaceae family consists of approximately 59 genera,with *Aglaia* being the largest among them. This family is primarily found in tropical regions but is also present in some temperate areas. Most species inhabit lowland tropical rainforests on dry land, though the family is also found on rocky shores and in mangrove swamps (species of *Xylocarpus*), freshwater swamp forest in Borneo (*Sandoricum borneense* Miq. and *Chisocheton amabilis* Miq.), drier forests, woodlands, and open savannas. They are poorly represented at higher altitudes, although some species of *Dysoxylum* and *Toona sinensis* (A.Juss.) M.Roem. are occasionally prominent in lower montane forest in Asia and *Ruagea* spp. in America. *Walsura monophylla* Elmer ex Merr. is limited to ultramafic soil in the Philippines. Meliaceae species are commonly found in secondary vegetation (*Toona* spp.), with some species becoming invasive weeds [46, 47].

## Phytochemistry

### Overview of nitrogen-containing compounds isolated from Meliaceae Family

**Fig. 1:**
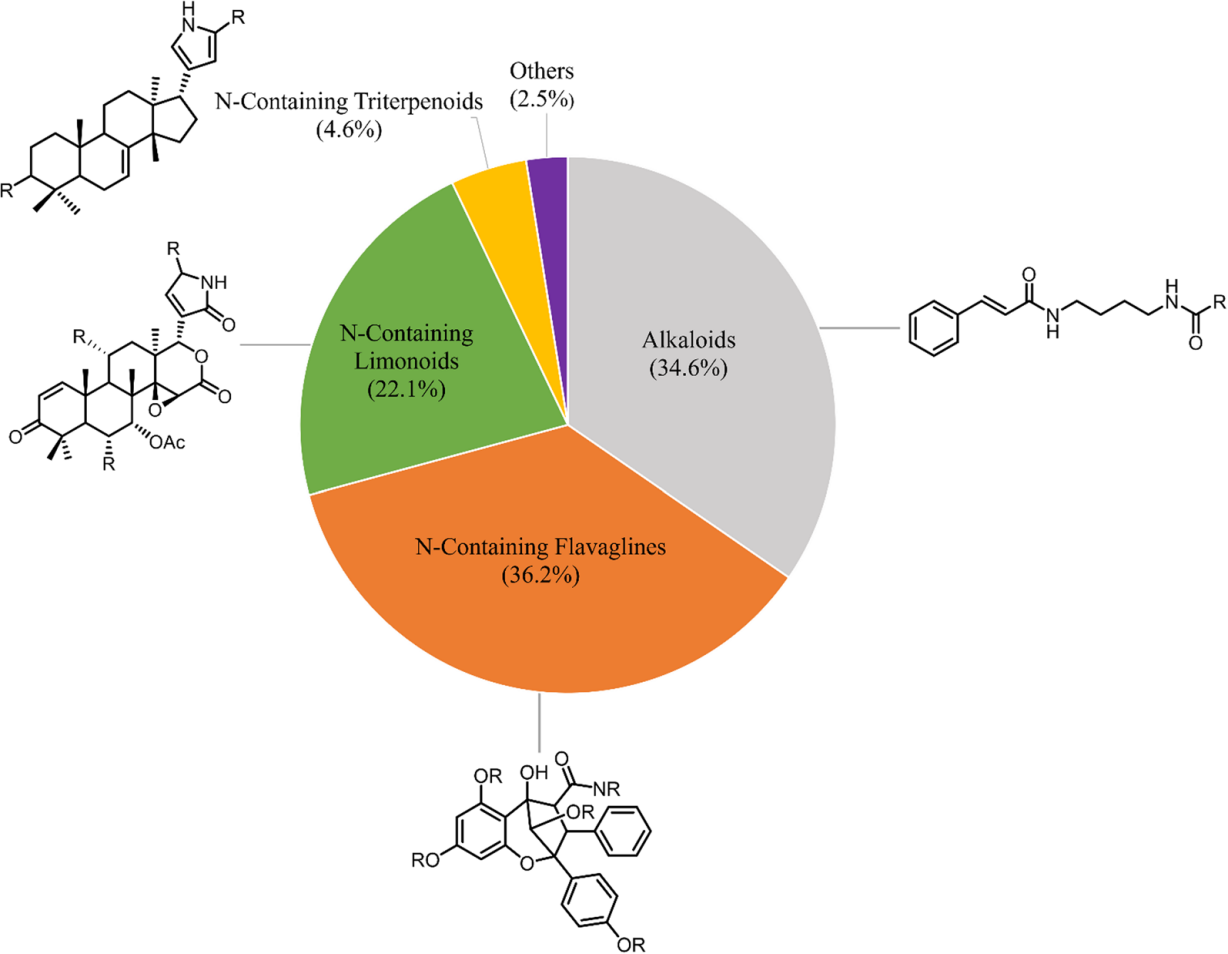
The distribution by groups of nitrogen-containing compounds from the Meliaceae family and their majors compounds

Based on scientific articles published from 1979 to 2024, a total of 326 nitrogen-containing compounds were obtained from the leaves, stems, stem barks, bark, roots, twigs, fruits, and seeds of plants in the Meliaceae family. The parts of the plant most frequently reported as source materials for isolation were the leaves followed by stem bark, roots, twigs, seeds, and fruit, with flowers being the least reported. Grouping the compounds according to structural class, nitrogen-containing flavaglines are the largest natural products, with a total of 118 compounds (36.2%), followed by alkaloids (34.6%), limonoids (22.1%), triterpenoids (4.6%), and others (2.5%), see Fig. 1.

### Alkaloids

Alkaloids typically contain one or more nitrogen atoms, usually in the form of primary, secondary, or tertiary amines, although quaternary amines are also present. For example, some alkaloids are essentially neutral, such as those with nitrogen in an amide function [48].

**Fig. 2:**
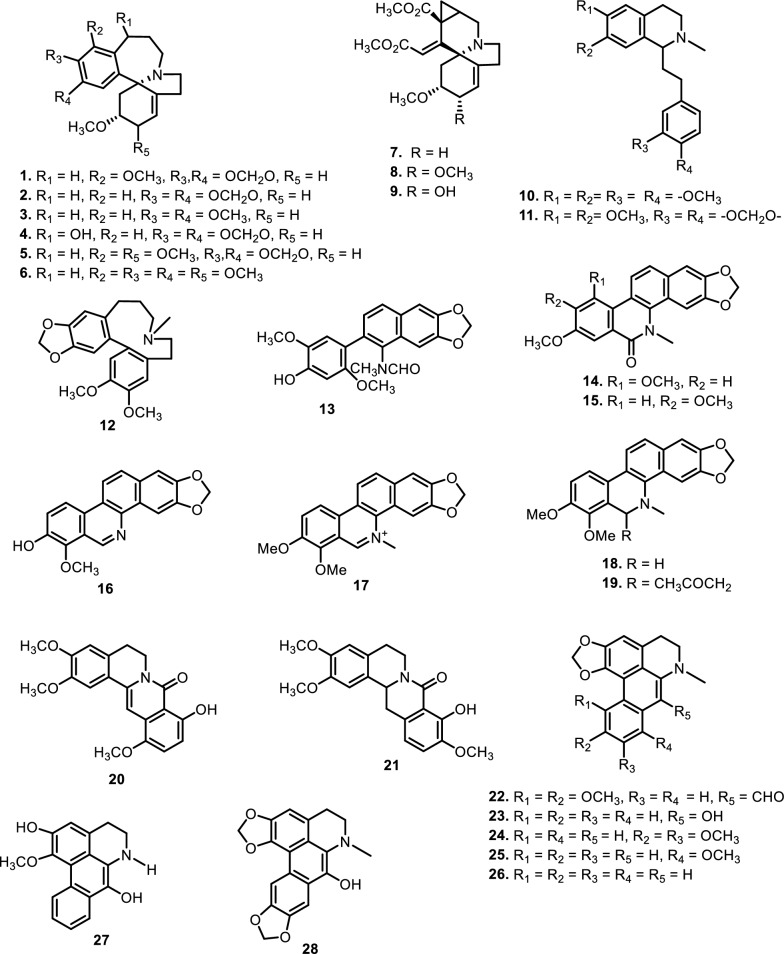
The chemical structures of compounds **1**–**28**

**Fig. 3:**
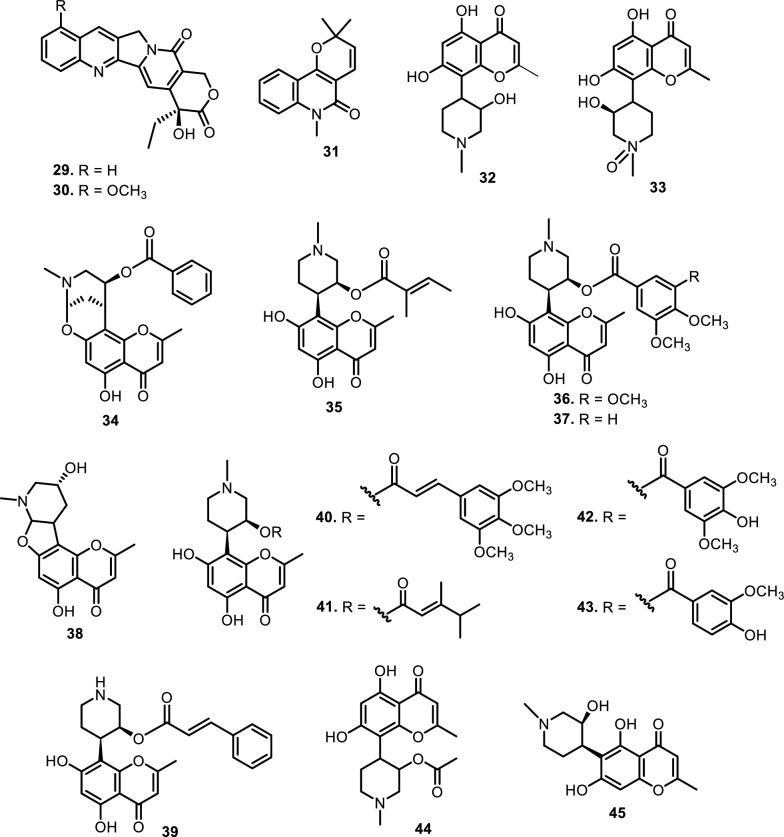
The chemical structures of compounds **29**–**45**

Different classes of alkaloids have been reported from the plants belonging to Meliaceae family. These include isoquinoline, quinoline, chromone, amide, carbazole, pyrrole, piperidine, indole, pyrazine, and β-carboline alkaloids. A summary of the number and types of alkaloids obtained from Meliaceae family is presented in Table 1, while Figs. 2 and 3 show the structures of the compounds.

**Table 1:** Alkaloids from Meliaceae family

No	Compounds	Species	Plant part	Biological activity	References
(1) Isoquinoline alkaloids					
(1.1) Homoerythrina alkaloids					
1	3-*epi*-18-methoxyschelhammericine (Dyshomerythrine)	*Dysoxylum lenticellare*	Leaves Stem	Not reported *B. glabrata* (LC_50_: 20 ppm)	[41] [50]
2	3-epischelhammericine	*D. lenticellare*	Leaves	*B. glabrata* (100% mortality at 10 ppm	[42]
				Not reported	[49]
3	2,7-dihydrohomoerysotrine	*D. lenticellare*	Leaves	*B. glabrata* (100% mortality at 8 ppm)	[42]
				Not reported	[49]
			Stem	Not reported	[50]
4	3-*epi*-12-hydroxyschelhammericine	*D. lenticellare*	Leaves	*B. glabrata* (100% mortality at 10 ppm)	[42]
5	3-*epi*-2,18-dimethoxy-schelhammericine	*D. lenticellare*	Stem	*B. glabrata* (LC_50_: 17.0 ppm)	[41]
				Not reported	[50]
6	2α-methoxycomosivine	*D. lenticellare*	Stem	Not reported	[51]
7	Lenticellarine	*D. lenticellare*	Leaves	*B. glabrata* (LC_50_: 40 ppm)	[43]
			Stem	Not reported	[41, 50]
8	2α-methoxylenticellarine	*D. lenticellare*	Stem	Not reported	[51]
9	2α-hydroxylenticellarine	*D. lenticellare*	Stem	Not reported	[51]
(1.2) Phenethylisoquinoline					
10	Homolaudanosine	*D. lenticellare*	Leaves	*B. glabrata* (100% mortality at 10 ppm)	[42]
				Not reported	[49]
11	Dysoxyline	*D. lenticellare*	Leaves	*B. glabrata* (100% mortality at 10 ppm)	[42]
				Not reported	[49]
(1.3) Dibenz[d,f]azecine					
12	Dysazecine	*D. lenticellare*	Leaves	*B. glabrata* (50% mortality at 20 ppm)	[42]
				Not reported	[49]
(1.4) Benzophenanthridine					
13	Turraeanthin A	*Turraeanthus africanus*	Stem bark	Not reported	[52]
14	Turraeanthin B	*T. africanus*	Stem bark	Not reported	[52]
15	Oxynitidine	*T. africanus*	Stem bark	Not reported	[52]
16	Decarine	*T. africanus*	Stem bark	Not reported	[52]
17	Chelerythrine	*Xylocarpus granatum*	Root bark	Not reported	[53]
18	Dihydrochelerythrine	*X. granatum*	Root bark	Not reported	[53]
19	Acetonyldihydrochelerythrine	*X. granatum*	Root bark	Not reported	[53]
(1.5) Oxoprotoberberine					
20	Amocurine A	*Amoora cucullata*	Roots	NCI-H187 (IC_50_: 20.5 ± 0.2 μM); KB and MCF-7 (inactive)	[28]
21	Amocurine B	*A. cucullata*	Roots	NCI-H187 ± 0.5 μM); KB and MCF-7 (inactive)	[28]
(1.6) Aporphine					
22	Amocurine C	*A. cucullata*	Leaves	Not reported	[45]
			Roots	KB (IC_50_: 3.5 ± 0.6 μM); MCF-7 (IC_50_: 4.2 ± 1.4 μM); and NCI-H187 (IC_50_: 6.7 ± 0.1 μM)	[28]
23	Amocurine D	*A. cucullata*	Stems	*S. aureus* and *B. subtilis* (MIC: 3.1 μg/mL)	[44]
			Leaves	*P. falciparum* strain K1 (IC_50_: 3.52 μM)	[45]
24	Dehydrodicentrine	*A. cucullata*	Roots	KB (IC_50_: 9.3 ± 0.8 μM); MCF-7 (IC_50_: 10.1 ± 0.2 μM); and NCI-H187 (IC_50_: 8.5 ± 0.5 μM)	[28]
			Leaves	Not reported	[45]
25	Stephanine	*A. cucullata*	Roots	NCI-H187 (IC_50_: 30.4 ± 1.1 μM); KB and MCF-7 (inactive)	[28]
			Leaves	Not reported	[45]
26	Roemerine	*A. cucullata*	Roots	NCI-H187 (IC_50_: 25.2 ± 0.8 μM); KB and MCF-7 (inactive)	[28]
			Leaves	Not reported	[45]
27	Amocurine E	*A. cucullata*	Stems	*B. subtilis* (MIC: 3.1 μg/mL)	[44]
28	Amocurine F	*A. cucullata*	Leaves	*P. falciparum* strain K1 (IC_50_: 1.84 μM)	[45]
(2) Quinoline alkaloids					
29	Camptothecin (CPT)	*Dysoxylum binectariferum*	Barks	Not reported	[54]
30	9-methoxy CPT	*D. binectariferum*	Barks	Not reported	[54]
(3) Quinolone alkaloid					
31	*N*-Methylflindersine	*Xylocarpus granatum*	Root barks	Not reported	[53]
(4) Chromone alkaloids					
32	Rohitukine	*Amoora rohituka*	Leaves and stem	Not reported	[32]
		*Dysoxylum acutangulum*	Leaves	HL-60 (IC_50_ 7.5 μM) and HCT-116 (8.8 μM)	[55]
		*Dysoxylum binectariferum*	Stem barks	SKOV3 (20 μg/mL), T47D (50 μg/mL), MDAMB 273 (3 μg/mL), NCI/ADR-RES (2.8 μg/mL), and MCF-7 (15 μg/mL)	[56]
			Stem barks	Not reported	[33, 57, 58]
			Fruit	TNF-α (50%) and IL-6 (82%) at 5 μM	[39]
33	Rohitukine-N-oxide	*D. binectariferum*	Stem barks	Not reported	[33, 58]
34	Chrotacumine A	*D. acutangulum*	Leaves	HL-60 (IC_50_ > 10 μM)	[55]
35	Chrotacumine B	*D. acutangulum*	Leaves	HL-60 (IC_50_ > 10 μM)	[55]
36	Chrotacumine C	*D. acutangulum*	Leaves	HL-60 (IC_50_ > 10 μM)	[55]
37	Chrotacumine D	*D. acutangulum*	Leaves	HL-60 (IC_50_ > 10 μM)	[55]
38	Chrotacumine E	*D. acutangulum*	Leaves	Tyrosinase inhibition activity (29.2% at 100 μg/mL)	[59]
		*D. binectariferum*	Seeds	TNF-α (33%) and IL-6 (16%) at 10 μM	[39]
39	Chrotacumine F	*D. acutangulum*	Leaves	Tyrosinase inhibition activity (25.8% at 100 μg/mL)	[59]
40	Chrotacumine G	*D. acutangulum*	Barks	Osteoclast differentiation inhibitory activity (IC_50_: 9.8 μM)	[60]
41	Chrotacumine H	*D. acutangulum*	Barks	Not reported	[60]
42	Chrotacumine I	*D. acutangulum*	Barks	Not reported	[60]
43	Chrotacumine J	*D. acutangulum*	Barks	Osteoclast differentiation inhibitory activity (IC_50_: 15.1 μM	[60]
44	Chrotacumine K	*D. binectariferum*	Fruits	TNF-α (81%) and IL-6 (20%) at 10 μM Non-toxic to THP-1 cells up to 40 μM	[39]
45	Dysoline	*D. binectariferum*	Stem barks	HT1080 (IC_50_: 0.21 μM) TNF-α (47%) and IL-6 (83%) at 0.1 μM Colo205, HCT116, NCIH322, A549, MOLT-4, and HL60 (IC_50_ ≥ 10 μM) Mild inhibiton (25%) of PKB-β at 0.5 μM, CDK2/A, CDK9/T1, Aurora A, Aurora B, AMPK (hum), CK1γ2, CK1δ, DYRK1A, IGF-1R, IR, VEGFR1 kinases (inactive)	[33]
(5) Amide alkaloids					
(5.1) Monoamide					
46	Tenucaulin A	*Aglaia tenuicaulis*	Leaves	Not reported	[61]
47	Isotenucaulin A	*A. tenuicaulis*	Leaves	Not reported	[61]
48	Tenucaulin B	*A. tenuicaulis*	Leaves	Not reported	[61]
49	Aglatenin	*A. tenuicaulis*	Leaves	Not reported	[61]
50	Tenaglin	*A. tenuicaulis*	Stem barks	Not reported	[61]
51	Caulitenin	*A. tenuicaulis*	Stem barks	Not reported	[61]
52	Aglatenol	*A. tenuicaulis*	Leaves	Not reported	[61]
53	Aglamide D	*Aglaia edulis*	Leaves	Lu1, LNCaP, MCF-7 and HUVEC (inactive)	[62]
(5.2) Bisamide					
54	Pyrrolotenin	*A. tenuicaulis*	Leaves	Not reported	[61]
55	Secopyrrolotenin	*A. tenuicaulis*	Leaves	Not reported	[61]
56	Secoisoodorinol	*Aglaia spectabilis*	Leaves	Not reported	[61]
57	Secoisopiriferinol	*A. spectabilis*	Leaves	Not reported	[61]
58	Aglamide A	*A. edulis*	Leaves	Lu1, LNCaP, MCF-7, and HUVEC (inactive)	[62]
59	Aglamide B	*A. edulis*	Leaves	Lu1, LNCaP, MCF-7, and HUVEC (inactive)	[62]
60	Aglamide C	*A. edulis*	Twigs	Lu1, LNCaP, MCF-7, and HUVEC (inactive)	[62]
61	Perviridamide	*Aglaia perviridis*	Twigs	Inhibitory in RAW 264.7 cells (IC_50_: 65.3 μg/mL)	[63]
62	4-hydroxypyramidatine	*A. perviridis*	Twigs	Inhibitory in RAW 264.7 cells (IC_50_: 83.4 μg/mL)	[63]
			Leaves	Not reported	[64]
63	Pyramidatine	*A. perviridis*	Twigs	Inhibitory in RAW 264.7 cells (inactive)	[63]
			Leaves	Not reported	[64]
		*Aglaia pyramidata*	Leaves	Not reported	[65]
		*Aglaia foveolata*	Leaves	Not reported	[66]
		*Aglaia gracilis*	Leaves	Not reported	[67]
		*Aglaia andamanica*	Leaves	Not reported	[68]
		*Aglaia forbesii*	Leaves	Not reported	[69]
64	*N*-(4-(2-(4-hydroxyphenyl) acetamido)butyl)cinnamamide	*A. perviridis*	Leaves	Not reported	[64]
65	Hemileptaglin	*Aglaia leptantha*	Stem barks	Not reported	[168]
66	Agleptin	*A. leptantha*	Stem barks	Not reported	[168]
67	Isoagleptin	*A. leptantha*	Leaves	Not reported	[168]
68	Leptanthin	*A. leptantha*	Leaves and stem barks	Not reported	[168]
69	Aglaithioduline (leptagline)	*A. edulis*	Leaves	HSV types 1 and 2 (< 50% reduction of plaque formation at 20 μg/mL)	[70]
		*A. leptantha*	Stem barks	Not reported	[168]
70	Aglaiduline (aglanthine)	*A. edulis*	Leaves	HSV types 1 and 2 (inactive)	[70]
		*A. leptantha*	Stem barks	Not reported	[168]
71	Aglaidithioduline	*A. edulis*	Leaves	HSV types 1 and 2 (< 50% reduction of plaque formation at 20 μg/mL)	[70]
72	Secoodorine	*A. gracilis*	Leaves	Not reported	[67, 169]
73	Secopiriferine	*A. gracilis*	Leaves	Not reported	[67]
74	Piriferine	*Aglaia pirifera*	Leaves	Not reported	[71]
		*A. pyramidata*	Leaves	KB-V1 (ED_50_: 10 μg/mL) and KB-V1* (ED_50_: 8.5 μg/mL) BCA-1, HT-1080. LUC-1, MEL-2, COL-1, KB, A-431, LNCaP, and ZR-75 (inactive)	[65]
		*Aglaia elaeagnoidea*	Leaves	Not reported	[74]
		*A. gracilis*	Leaves	Not reported	[67]
		*Aglaia elliptifolia*	Leaves	Not reported	[72]
		*Aglaia testicularis*	Leaves	Not reported	[73]
		*Aglaia odorata*	Leaves and twigs	Not reported	[29]
75	Piriferinol	*A. elaeagnoidea*	Leaves	*S. littoralis* (inactive, LC_50_ and EC_50_ > 250 μg/g fr.wt)	[74]
76	Edulimide	*A. edulis*	Leaves	*S. littoralis* (inactive, LC_50_ and EC_50_ > 250 μg/g fr.wt)	[74]
77	Odorine	*A. odorata*	Leaves	Not reported	[75]
				Inhibitory in RAW 264.7 cells (IC_50_: 8.6 μg/mL)	[40]
			Leaves and twigs	Not reported	[29]
		*Aglaia roxburghiana*	Leaves	Not reported	[30]
		*A. pirifera*	Leaves	Not reported	[71]
		*A. pyramidata*	Leaves	KB-V1* (ED_50_: 6.4 μg/mL) BCA-1, HT-1080. LUC-1, MEL-2, COL-1, KB, KB-V1, A-431, LNCaP, and ZR-75 (inactive)	[65]
		*Aglaia argantea*	Leaves	Not reported	[76]
		*Aglaia laxiflora*	Leaves	Not reported	[77]
		*Aglaia elliptica*	Leaves	Not reported	[78]
		*A. gracilis*	Leaves	Not reported	[67]
		*Aglaia oligophylla*	Leaves	Not reported	[79]
78	5'-*epi*-odorine	*A. pyramidata*	Leaves	KB-V1* (ED_50_: 4.2 μg/mL) BCA-1, HT-1080. LUC-1, MEL-2, COL-1, KB, KB-V1, A-431, LNCaP, and ZR-75 (inactive)	[65]
79	Aglairubine	*Aglaia rubiginosa*	Leaves	Not reported	[81]
		*A. spectabilis*	Leaves	Not reported	[61, 80]
80	Grandiamide B	*Aglaia grandis*	Leaves	Not reported	[82]
81	Grandiamide C	*A. grandis*	Leaves	Not reported	[82]
82	Grandiamide D	*Aglaia gigantea*	Leaves	Not reported	[83]
83	Gigantamide A	*A. gigantea*	Leaves	Not reported	[83]
		*A. perviridis*	Combination of the leaves, twigs, and fruits	HT-29 (ED_50_: 0.021 μM) NF-κB (inactive, ED_50_ > 20 μM)	[84]
84	Dasyclamide	*Aglaia dasyclada*	Leaves	Not reported	[84]
		*Aglaia gigantea*	Leaves	Not reported	[83]
		*Amoora cucullata*	Leaves	TRAIL resistance-overcoming activity (inactive)	[86]
				Not reported	[85]
85	Cucullamide	*A. cucullata*	Leaves	Not reported	[86]
86	Aglaianine	*Aglaia abbreviata*	Leaves	Not reported	[170]
87	Aglaiamide A	*A. perviridis*	Leaves	Not reported	[64]
				Inhibitory in RAW 264.7 cells (IC_50_: 7.4 μg/mL)	[26]
88	Aglaiamide O	*A. perviridis*	Leaves	Inhibitory in RAW 264.7 cells (IC_50_: 19.5 μg/mL)	[26]
89	Aglaiamide P	*A. perviridis*	Leaves	Inhibitory in RAW 264.7 cells (IC_50_: 12.3 μg/mL)	[26]
90	Elliptinol	*A. elliptifolia*	Leaves	HL-60 (ED_5_: 32.1 μg/mL) and P-388 ((ED_50_: 3.62 μg/mL) A549, HT-29, and KB (inactive)	[72]
91	Dehydroodorin	*A. elliptifolia*	Leaves	Not reported	[72]
		*Aglaia formosana*	Leaves	P-388 (ED_50_: 3.86 μg/mL)	[87]
92	Eximiamide A	*Aglaia eximia*	Barks	P-388 (IC_50_: 7.6 μg/mL)	[88]
93	Eximiamide B	*A. eximia*	Barks	P-388 (IC_50_: 8.5 μg/mL)	[88]
94	Aurantiamide acetate	*Aglaia ouangliensis*	Leaves, twigs	Inhibitory in RAW 264.7 cells (at 40 and 80 μM)	[89]
95	Benzenepropanamide	*A. ouangliensis*	Leaves, twigs	Inhibitory in RAW 264.7 cells (at 20, 40 and 80 μM)	[89]
96	2'-*epi*-odorine	*A. oligophylla*	Leaves	Not reported	[79]
97	Roxburghilin	*A. roxburghiana*	Leaves	Not reported	[30]
98	Odorinol	*A. odorata*	Leaves and twigs	P-388 (T/C: 136%)	[90]
				Not reported	[29]
			Leaves	Not reported	[77]
				Inhibitory in RAW 264.7 cells (IC_50_: 50.2 μg/mL)	[77]
		*A. laxiflora*	Leaves	Not reported	[77]
		*A.testicularis*	Leaves	Not reported	[73]
99	Agladorin A	*A. odorata*	Leaves and twigs	α-Glucosidase, BuChE and PTP1B inhibitory activities (inactive at 50 μg/mL	[29]
100	(−)-odorinal	*A. odorata*	Leaves and twigs	α-Glucosidase, BuChE and PTP1B inhibitory activities (inactive at 50 μg/mL	[29]
101	Agladorin B	*A. odorata*	Leaves and twigs	α-Glucosidase, BuChE and PTP1B inhibitory activities (inactive at 50 μg/mL	[29]
(5.3) Poliamide					
102	Chisitine 1	*Chisocheton weinlandii*	Leaves	Not reported	[91]
103	Chisitine 2	*C. weinlandii*	Leaves	Not reported	[91]
(6) Carbazole alkaloids					
104	Ekeberginine	*Ekebergia senegalensis*	Stem barks	Not reported	[92]
105	*N*-Methylekeberginine	*E. senegalensis*	Stem barks	Not reported	[92]
106	Indizoline	*Ekebergia senegalensis*	Stem barks	Not reported	[92]
107	Heptaphylline	*E. senegalensis*	Stem barks	Not reported	[92]
(7) Pyrrole alkaloid					
108	β-D-glucopyranos-1-yl *N*-methylpyrrole-2-carboxylate	*A. odorata*	Roots	A549, MCF-7, SW480, SMMC-7721 and HL-60 (inactive)	[93]
(8) Piperidine alkaloid					
109	1-acetyl-4,4-bis[4-(3-bromopropoxy)-3,5-dimethoxyphenyl] piperidine	*Swietenia mahagoni*	Leaves	Not reported	[94]
(9) Indole alkaloid					
110	Methyl indole-3-carboxylate	*Melia azedarach* L	Leaves	Inhibitory in RAW 264.7 cell (IC_50_: 42.2 ± 2.7 μM) HL60, A549, AZ521, SK-BR-3 (inactive)	[95]
(10) Pyrazine alkaloid					
111	Xylogranatinin	*X. granatum*	Fruits	Not reported	[96]
		*Amoora ouangliensis*	Leaves and twigs	Not reported	[89]
(11) β-Carboline alkaloids					
112	4,8-Dimethoxy-1-vinyl-β-carboline	*M. azedarach*	Cortex	Inhibitory in RAW 264.7 cell (IC_50_:< 2 μM)	[97]
113	4-Methoxy-1-vinyl-β-carboline	*M. azedarach*	Cortex	Inhibitory in RAW 264.7 cell (IC_50_: 2.8 μM)	[97]

#### Isoquinoline alkaloids

**Fig. 4:**
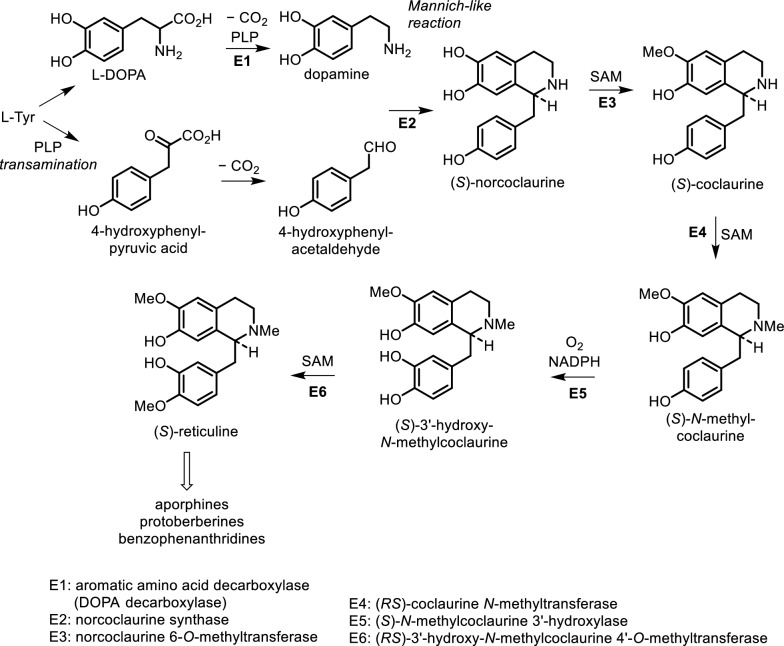
The biosynthetic pathways to several types of isoquinoline alkaloids from Meliaceae family

Isoquinoline alkaloids are derived from tyrosine and phenylalanine. These compounds are synthesized from the precursor 3,4-dihydroxyphenylethylamine (dopamine) via a reaction with an aldehyde or ketone. The product from the Mannich-like reaction is thus the trihydroxy alkaloid norcoclaurine, formed stereospecifically as the (*S*)-enantiomer. The tetrahydroxy substitution pattern is built up by further hydroxylation in the benzyl ring, though *O*-methylation (giving (*S*)-coclaurine) and *N*-methylation steps precede this. Eventually, (*S*)-reticuline, a pivotal intermediate to other alkaloids, is attained by *N*-methylation [48]. The biosynthetic pathways for several types of isoquinoline alkaloids from the Meliaceae family are shown in Fig. 4. In line with these biosynthetic pathways, a total of 28 isoquinoline alkaloids had been identified from different classes including homoerythrina, phenylethylisoquinoline, dibenz[d,f]azecine, benzophenanthridine, oxoprotoberberine, and aporphine alkaloids.

Among these classes, homoerythrina alkaloids represent a growing class of natural products, predominantly isolated from the *Dysoxylum* genus. These compounds are characterized by a tetracyclic C_17_ skeleton, which constitutes a structural expansion of the erythrinan-type alkaloid framework, particularly at the nitrogen-containing ring. The homoerythrina scaffold retains the fused aromatic and nitrogenous ring system common to *Erythrina* alkaloids but incorporates an additional carbon atom in the core structure, resulting in subtle yet significant differences in molecular flexibility and physicochemical properties. Specifically, the nine alkaloids investigated in this study, namely dyshomerythrine (**1**), 3-epischelhammericine (**2**), 2,7-dihydrohomoerysotrine (**3**), 3-epi-12-hydroxyschelhammericine (**4**), 3-epi-18-methoxyschelhammericine (**5**), 2α-methoxycomosivine (**6**), lenticellarine (**7**), 2α-methoxylenticellarine (**8**), and 2α-hydroxylenticellarine (**9**), belong to this class and can be categorized into two structural subtypes. Compounds **1–6** share a polycyclic backbone with a fused aromatic ring, a nitrogenous ring, and an oxygenated six-membered ring. Their structural variation arises from different substituents (methoxy, hydroxyl, and methylenedioxy) at positions C-2, C-12, C-16, C-17, and C-18. For instance, compound **1** features a methoxy group at C-18 and a methylenedioxy bridge at C-16 and C-17, whereas compound **2** lacks methoxy groups but retains the bridge. In compound **3**, the bridge is replaced by discrete methoxy groups, while compound **4** carries a hydroxyl at C-12. Methoxylation increases in compounds **5** and **6**, with compound **6** bearing four methoxy groups on the aromatic ring [42, 49]. In contrast, compounds **7–9** form a distinct subgroup, defined by a bicyclic lactam system, two methyl ester groups, a methoxy substituent at C-3, and variation at the 2α-position. Compound **7** bears a hydrogen, compound **8** a methoxy, and compound **9** a hydroxyl group at this position, altering polarity and hydrogen-bonding capacity. All nine compounds were isolated from *Dysoxylum lenticellare* Gillespie. Compounds **1–4** and **7** were obtained from the leaves, while **1–3**, **5–6**, and **7** were also found in the stem. Notably, compounds **8** and **9** were exclusive to the stem, indicating tissue-specific distribution [41, 50, 51].

Beyond the homoerythrina class, the phenethylisoquinoline class includes homolaudanosine (**10**) and dysoxyline (**11**), which are biosynthetically derived from the condensation of dopamine and phenylacetaldehyde. These compounds are characterized by a linear framework composed of a 1-benzylisoquinoline core, with a phenethyl side chain attached at the C-1 position of the isoquinoline. This scaffold permits a high degree of substitution, especially with methoxy and methylenedioxy groups, which significantly influence their physicochemical properties and molecular conformation [42, 50]. In addition, dibenz[d,f]azecine alkaloids, such as dysazecine (**12**), introduce unique rigid frameworks, which may contribute to their distinctive pharmacological profiles [42, 49]. Furthermore, Vardamides et. al. reported the presence of four benzophenanthridine-type alkaloids including turraeanthins A-B (**13–14**), oxynitidine (**15**), and decarine (**16**), which were obtained from the stem bark of *Turraeanthus africanus* (Welw. ex C.DC.) Pellegr [52]. Compound **13** bears a formyl group (–CHO) attached to the N-methylated nitrogen at position 7. It also features hydroxyl and methoxy substituents at C-10 and C-11, respectively, which enhance hydrogen bonding and electron density. Additionally, the dioxolane ring spanning positions 2 and 3 contributes to conformational rigidity and increased lipophilicity. Compounds **14** and **15** are oxo-derivatives with two methoxy and the methylenedioxy at C-2 and C-3. They are structural isomers differing only in the position of the methoxy groups (C-11 and C-12). These changes may influence resonance stabilization and electron distribution within the aromatic system. Compound **16** is a fully aromatic benzophenanthridine with a hydroxyl at C-10 and a methoxy at C-9. It lacks N-substitution, rendering it more reactive toward electrophilic substitutions or oxidative transformations. Similarly, the root barks of *Xylocarpus granatum* J.Koenig contained three compounds including, chelerythrine (**17**), dihydrochelerythrine (**18**), and acetonyldihydrochelerythrine (**19**) [53]. Compound **17** represents the quaternary benzophenanthridinium form, where the nitrogen atom is methylated and carries a permanent positive charge. Compound **18** is the parent dihydrobenzophenanthridine with two methoxy groups at C-9 and C-10 and a free secondary nitrogen. Compound **19** is a substituted derivative of compound **18**, bearing an acetonyl side chain at the aromatic ring. This side chain increases the molecule’s steric bulk and enhances lipophilicity.

Moving on to the oxoprotoberberine class, amocurines A (**20**) and B (**21**) are newly characterized alkaloids belonging to the oxoprotoberberine class, featuring a distinctive tetracyclic isoquinoline-derived scaffold. Structurally, these compounds differ in their substitution patterns, particularly in the number and position of methoxy groups on the aromatic rings. Both compounds were isolated from *Amoora cucullata* Roxb. (syn. *Aglaia cucullata* (Roxb.) Pellegr.). Moreover, six aporphine alkaloids, namely amocurines C-D (**22–23**), dehydrodicentrine (**24**), stephanine (**25**), roemerine (**26**), and amocurine E (**27**), were also isolated from the same plant [28, 44, 45]. These compounds differ by their substitution patterns on the aromatic rings, which modulate physicochemical properties such as polarity, electronic distribution, and lipophilicity. Compound **26** is the simplest analog, lacking any ring substituents, rendering it more hydrophobic. [45] reported the presence of a novel aporphine alkaloid, namely amocurine F (**28**), from the leaves of *Amoora cucullata* Roxb., along with five known compounds **22–26**. Uniquely, Amocurine F features two methylenedioxy bridges: one on ring A (positions 1 and 2) and another on ring D (positions 9 and 10), creating a more conformationally constrained and electron-rich aromatic system.

#### Quinoline and quinolone alkaloids

Quinoline and quinolone alkaloids represent a structurally and biologically significant subclass within the Meliaceae family, albeit with a limited number of identified representatives. To date, only two quinoline alkaloids, camptothecin (**29**) and 9-methoxy camptothecin (**30**), have been isolated from the ethanolic extract of the bark of *Dysoxylum binectariferum* Hook.f. ex Bedd. [54]. Camptothecin is a well-known cytotoxic agent that serves as the precursor for clinically approved chemotherapeutic drugs such as topotecan and irinotecan. The occurrence of camptothecin in *Dysoxylum binectariferum* underscores the chemotaxonomic significance of this species within Meliaceae. Interestingly, this alkaloid has been exclusively found in the stem bark of *D. binectariferum*, suggesting a highly localized biosynthetic pathway. The presence of a methoxy substituent at C-9 in compound **30** increases its lipophilicity and may alter the electronic distribution across the aromatic system, potentially influencing its chemical reactivity and solubility. In addition, a quinolone alkaloid, *N*-methylflindersine (**31**), has been identified in the root bark of *Xylocarpus granatum* J. Koenig, further expanding the alkaloidal diversity within Meliaceae [53].

#### Chromone alkaloids

Expanding on the alkaloidal diversity, chromone alkaloids have been reported exclusively from the genus *Dysoxylum* within the Meliaceae family. A total of 14 chromone alkaloids have been identified, and these compounds exhibit a broad spectrum of bioactivities, underscoring their pharmaceutical potential. The first chromone alkaloid reported from Meliaceae was rohitukine (**32**), which was isolated in 1979 from the leaves and stem of *Amoora rohituka* Wight & Arn (syn. *Aphanamixis polystachya* (Wall.) Parker) [32]. This compound has also been reported in the leaves of *Dysoxylum acutangulum* Miq [55]. and stem bark of *Dysoxylum binectariferum* (Roxb.) Hook.f. ex Bedd. [33, 56, 57]. According to subsequent phytochemical studies, rohitukine is a taxonomically significant metabolite predominantly found in a limited number of Meliaceae species, particularly within the genera *Amoora* and *Dysoxylum*. This specificity makes rohitukine a valuable chemical marker for the family. [58] reported the presence of compound **32** from the stem bark of *Dysoxylum binectariferum* (Roxb.) Hook. f. ex Bedd., along with its oxidized analogue, rohitukine *N*-oxide (**33**), which features an additional N → O group that increases polarity. Another study reported that the leaves of *Dysoxylum acutangulum* Miq. yielded four new chromone alkaloids, including chrotacumines A-B (**34–35**) [55] and chrotacumines E–F (**38–39**) [59]. Meanwhile, chrotacumines C-D (**36–37**), isolated from the bark of *Dysoxylum acutangulum* Miq., feature extended aromatic substitution with methoxy groups that modulate electronic distribution and lipophilicity [55]. [60] isolated four chromone alkaloids chrotacumines G-J (**40–43**) from the bark of *Dysoxylum acutangulum* Miq. Adding to this, [39] investigated the fruits of *Dysoxylum binectariferum* Hook f., which led to the isolation of one novel chromone alkaloid, chrotacumine K (**44**) with rohitukine (**32**) and chrotacumine E (**38**). Dysoline (**45**) was also isolated from the stem bark, along with rohitukine *N*-oxide (**33**) [33].

#### Amide alkaloids

Amide alkaloids constitute another major class within the Meliaceae family, with sulfur-containing and bisamide derivatives being particularly prominent. *Aglaia tenuicaulis* Hiern has yielded several sulfur-containing monoamide-esters, including tenucaulin A (**46**), isotenucaulin A (**47**), tenucaulin B (**48**), and aglatenin (**49**)*.* Further exploration of *A. tenuicaulis* stem bark led to the discovery of two new monoamide-esters: tenaglin (**50**) and caulitenin (**51**), which are characterized by phenylacryloyl and aryl ester motifs, respectively, providing greater aromatic character and conjugation. Additionally, the leaf extract yielded one amide alcohol, aglatenol (**52**). The bisamide class is exemplified by compounds such as pyrrolotenin (**54**) and secopyrrolotenin (**55**), also isolated from the leaves of *A. tenuicaulis*. In a similar context, two structurally distinct bisamides, secoisoodorinol (**56**) and secoisopiriferinol (**57**), were identified in *Aglaia spectabilis* (Miq.) Jain & Bennet [61]. Other compounds found in *Aglaia* genus such as aglamide D (**53**), a new monoamide, along with three bisamides, aglamides A–C (**58–60**), were isolated from the leaves and twigs of Aglaia edulis (Roxb.) Wall. Compounds **58** and **59**, obtained from the leaves, are sulfur-containing compounds, with **59** representing the first sulfonyl-bearing alkaloid reported from the genus Aglaia. Compound **60**, isolated from the twigs, features an isobutyl group on the amide nitrogen, enhancing hydrophobicity and steric bulk. Structurally, **58** carries a methylthio group, while **59** has a more polar methylsulfonyl substituent on the cinnamoyl moiety [62]. All three compounds possess a conjugated phenyl double bond system, promoting planarity and potential π–π interactions with biological targets.

Expanding on the bisamide diversity, three related compounds such as perviridamide (**61**), 4-hydroxypyramidatine (**62**), and pyramidatine (**63**) are structurally related compounds characterized by their bisamide framework. Compound **61** only differs by one methylene group from the other two compounds. **62** is distinguished from **63** by the presence of a hydroxyl group at fourth position. Compounds **61–63** were isolated from the chloroform extract of the twigs of *Aglaia perviridis* Hiern [63]. Additionally, compound **62** and **63**, along with *N*-(4-(2-(4-hydroxyphenyl) acetamido) butyl)-cinnamamide (**64**), were isolated from the leaves of *Aglaia perviridis* Hiern [64]. Compound **63** was extracted from the leaves of several other *Aglaia* species, such as *Aglaia pyramidata* Hance [65] and *Aglaia foveolata* Pannell [66], *Aglaia gracilis* A.C.Sm. [67], *Aglaia andamanica* Hiern [68] and *Aglaia forbesii* King [69].

Another six new amides have been isolated from the leaves and stem barks of *Aglaia leptantha* Miq., namely hemileptaglin (**65**), agleptin (**66**), isoagleptin (**67**), leptanthin (**68**), leptagline (**69**), and aglanthine (**70**). Specifically, compound **69** was identical to aglaithioduline, while compound **70** was structurally related to aglaiduline. These two compounds, along with aglaidithioduline (**71**), were isolated from the leaves of *Aglaia edulis* (Roxb.) Wall. [70]. Closely related analogs such as secoodorine (**72**), secopiriferine (**73**), and piriferine (**74**) were extracted from the leaves of *Aglaia gracilis* A.C.Sm. [67]. Additionally, compound **74** was also found in *Aglaia pyramidata* Hance [65], *Aglaia pirifera* Hance (syn. *Aglaia edulis* (Roxb.) Wall.) [71]*, Aglaia elliptifolia* Merr. [72]*, Aglaia testicularis* C.Y.Wu (syn*. Aglaia edulis* (Roxb.) Wall.) [73]*,* and *Aglaia odorata* Lour. [29]. *Aglaia elaeagnoidea* Benth. leaves contained two new bisamide derivatives, namely piriferinol (**75**) and edulimide (**76**) [74]. Odorine (**77)** was found in several other *Aglaia* species, such as *Aglaia odorata* Lour. [29, 40, 75]*, Aglaia roxburghiana* Miq. [30]*, Aglaia pirifera* Hance [71]*, Aglaia pyramidata* Hance [65], *Aglaia argentea* Blume [76]*, Aglaia laxiflora* Miq. [77]*, Aglaia elliptica* Blume [78]*, Aglaia gracilis* A.C.Sm. [67]*,* and *Aglaia oligophylla* Miq. [79]. Compound **75** is a hydroxylated analog of **74**, whereas **77** and 5’-epi-odorine (**78**) were epimers. The aglairubine (**79**) is a bisamide bearing a hydroxyl group at the terminus, which has been isolated from leaves of *Aglaia spectabilis* (Miq.) [61, 80] and *Aglaia rubiginosa* (Hiern) Pannel [81].

Furthermore, two putrescine bisamides, grandiamides B-C (**80–81**) were obtained from the leaves of *Aglaia grandis* Korth. [82], while grandiamide D (**82**), gigantamide A (**83**), and dasyclamide (**84**) were isolated from the leaves of *Aglaia gigantea* Pellegr. [83]*.* The leaves, twigs, and fruits of *Aglaia perviridis* Hiern also yielded compound **83** [27], while the leaves of *Aglaia dasyclada* F.C.How & T.C.Chen [84] and *Amoora cucullata* Roxb. yielded compound **84** [85, 86]. Additionally, one new bisamide derivative has been isolated and elucidated as cucullamide (**85**) [86]. The aglaianine (**86**) has been isolated from the leaves of *Aglaia abbreviata* C.Y.Wu, while aglaiamide A (**87**), aglaiamide O (**88**), and aglaiamide P (**89**) were obtained from the leaves of *Aglaia perviridis* Hiern [26]. Furthermore, two bisamides, namely elliptinol (**90**) and dehydroodorin (**91**) were derived from the leaves of *Aglaia elliptifolia* Merr. [72]. [87] also obtained **91** from the leaves of *Aglaia formosana* Hayata. [88] reported two novel bisamides, eximiamides A-B (**92–93**) from the leaves of *Aglaia eximia* Miq. Moreover, *Amoora* genus has been shown to produce bisamide compounds such as those isolated from *Amoora ouangliensis* (H.Lev.) C.Y.Wu barks, including aurantiamide acetate (**94**) and benzenepropanamide (**95**) [89]. *Aglaia oligophylla* Miq. leaves yielded 2'-*epi*-odorine (**96**) [79], while roxburghilin (**97**) and odorinol (**98**) were the first bisamides to be isolated from Meliaceae species [30, 75]. Compound **98** was also reported from *Aglaia odorata* Lour. leaves and twigs [29, 40, 90], *Aglaia laxiflora* Miq [77]. and *Aglaia testicularis* C.Y.Wu leaves [73]. A recent paper by [29] reported four bisamides, compound **98**, agladorin A (**99**), (−) odorinal (**100**), and agladorin B (**101**) from the leaves and twigs of *Aglaia odorata* Lour.

The leaves of *Chisocheton weinlandii* Harms were found to contain two polyamide alkaloids, chisitines 1 and 2 (**102–103**). These two compounds were elucidated by mass spectrometry in combination with tandem-mass spectrometry (MS–MS) and then chemically isolated. The chemical structures of compounds **102** and **103** share a common polyamide backbone but differ in the nature of their side chains and functional groups. Chisitine 1 (**102**) features a side chain with a methylthio group attached to the amide, which introduces both steric and electronic effects that may influence the compound’s solubility and reactivity. In contrast, chisitine 2 (**103**) has a similar polyamide core but lacks the methylthio group, instead incorporating a phenyl group attached to the amide nitrogen, which may alter its hydrophobicity and interaction with biological targets. Both compounds maintain an aromatic ring linked to the amide group, which likely contributes to their structural stability and potential bioactivity [91].

#### Carbazole alkaloids

Carbazole alkaloids are a distinct class of naturally occurring compounds characterized by a fused tricyclic system consisting of two benzene rings joined on either side of a pyrrole ring. These alkaloids exhibit significant biological activities, including cytotoxic, antimicrobial, and antioxidant properties, making them valuable in pharmacological research. To date, only four carbazole alkaloids have been identified within the Meliaceae family, all originating from the genus *Ekebergia*. These include ekeberginine (**104**), *n*-methylekeberginine (**105**), indizoline (**106**), and heptaphylline (**107**). The main structural difference between compounds **104** and **105** is the *N*-substitution, with **105** having a methyl group on the nitrogen atom, whereas **104** remains unsubstituted. Compound **106** also attaches methoxy and prenyl substituents but is distinguished by the position of its functional groups. Compound **107**, in contrast, incorporates a hydroxyl group at C-2 and an aldehyde at C-3, along with a prenyl side chain, further diversifying the substitution pattern of this class [92].

#### Pyrrole, piperidine, indole, pyrazine, and β-carboline alkaloid

Beyond amides, several other nitrogen-containing alkaloid classes, including pyrrole, piperidine, indole, pyrazine, and β-carboline alkaloids, have also been identified in various species of the Meliaceae family, reflecting the structural diversity within this plant group. Among these, the pyrrole alkaloid β-D-glucopyranos-1-yl-N-methylpyrrole-2-carboxylate (**108**) was isolated from the roots of *Aglaia odorata* Lour. [93], representing a rare sugar-conjugated derivative in the family. In contrast, a structurally more complex piperidine alkaloid, 1-acetyl-4,4-bis[4-(3-bromopropoxy)-3,5-dimethoxyphenyl]piperidine (**109**), was obtained from the methanolic extract of *Swietenia mahagoni* (L.) Jacq. Leaves [94], showcasing a substituted piperidine scaffold bearing brominated side chains. Among the limited indole-derived metabolites reported in the Meliaceae family, methyl indole-3-carboxylate (**110**) has been isolated from the leaves of *Melia azedarach* L. [95]. Another pyrazine alkaloid, xylogranatinin (**111**) was found for the first time in the fruits of *Xylocarpus granatum* J.Koenig [96]*,* and later detected in the leaves and twigs of *Amoora ouangliensis* (H.Lev.) C.Y.Wu [93]*.* This compound marks one of the few pyrazine representatives in the family and may hold ecological or defensive roles. Among the most biologically intriguing are* t*he β-carboline alkaloids, a class known for their neuropharmacological and cytotoxic properties, have also been reported from Meliaceae species. Notably, 4,8-dimethoxy-1-vinyl-β-carboline (**112**) and 4-methoxy-1-vinyl-β-carboline (**113**) have been reported by [97] from the cortex of *Melia azedarach* L. The structural difference between compounds **112** and **113** lies solely in the substituent at C-8, with compound **112** bearing a methoxy group, whereas compound **113** is unsubstituted at this position.

**Fig. 5:**
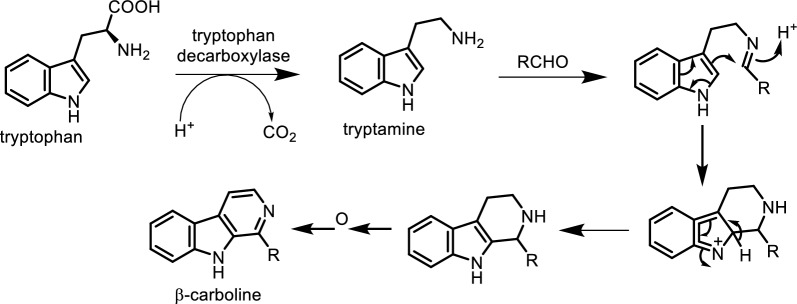
The biosynthetic pathways of β-carboline alkaloids

**Fig. 6:**
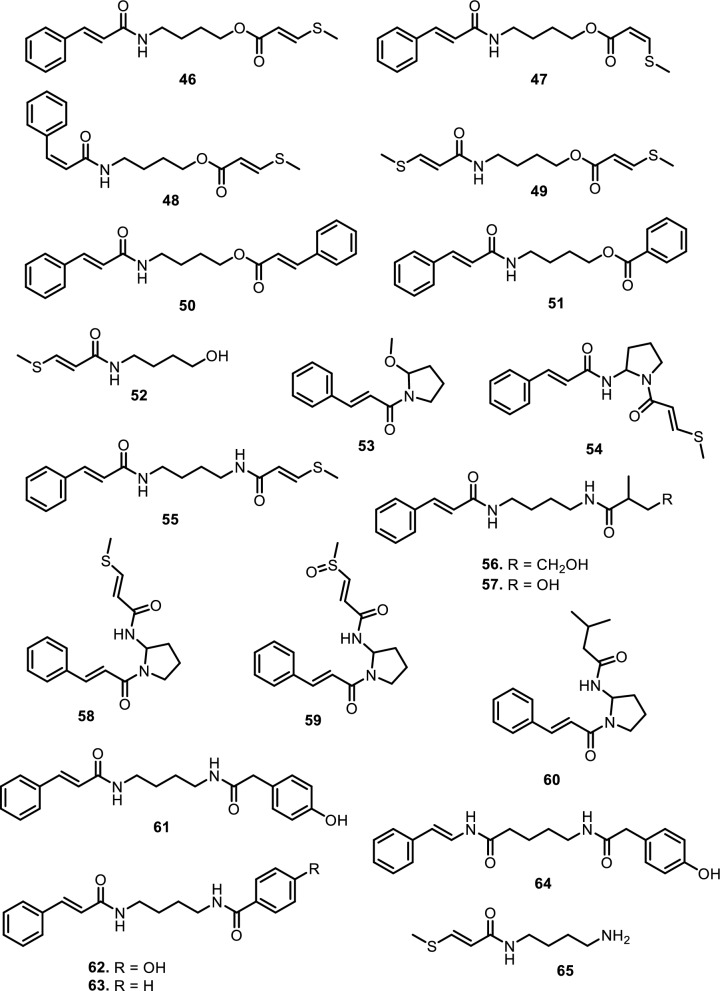
The chemical structures of compounds **46**–**65**

**Fig. 7:**
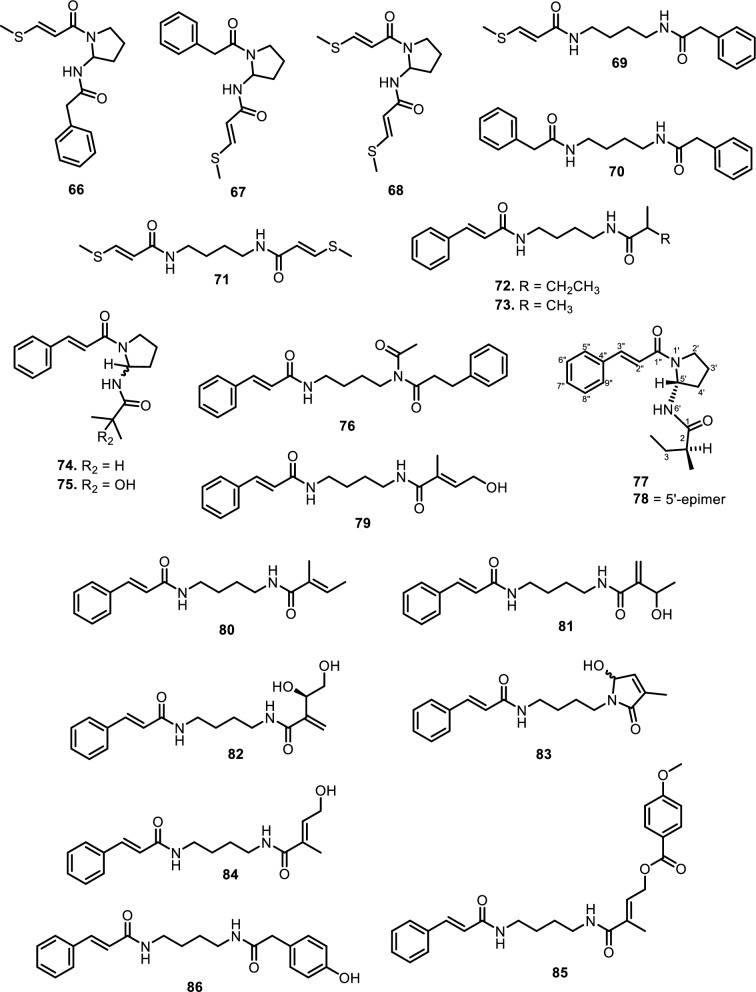
The chemical structures of compounds **66**–**86**

**Fig. 8:**
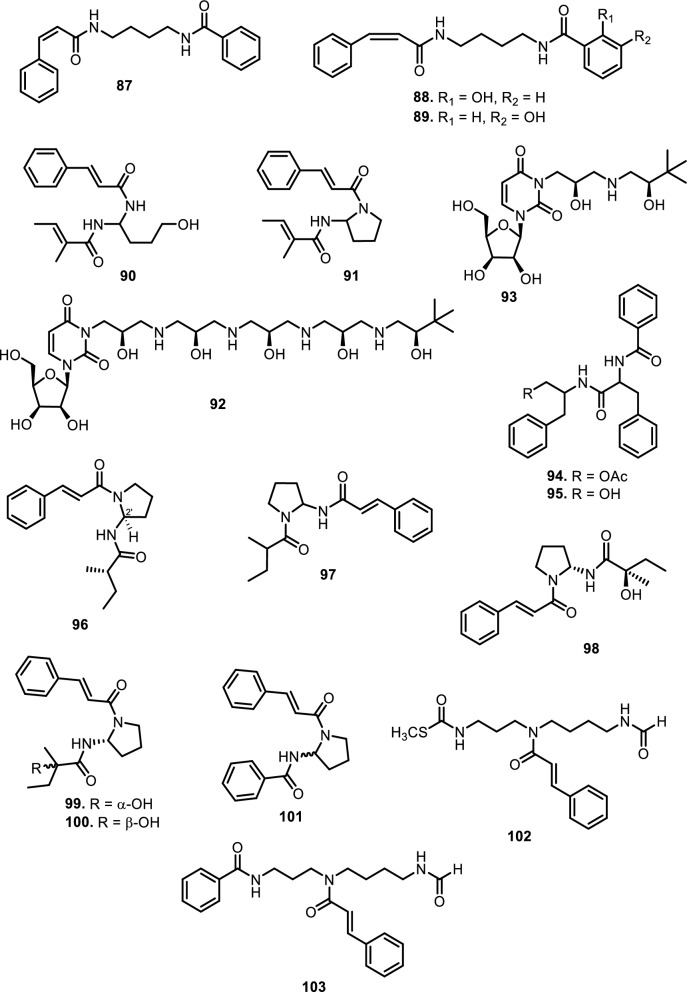
The chemical structures of compounds **87**–**103**

**Fig. 9:**
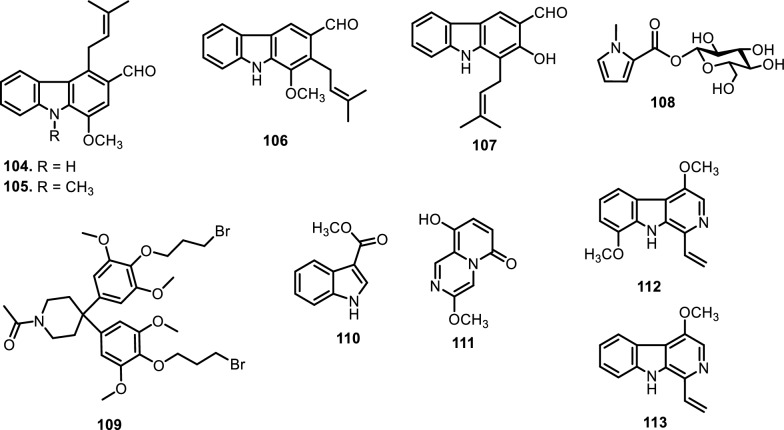
The chemical structures of compounds **104**–**113**

The biosynthesis of β-carboline alkaloids involves the condensation of tryptamine with a compound containing an aldehyde carbonyl group, resulting in an imine structure. Cyclization of the amine with the double bond on the indole forms a heterocyclic three-ring structure, which yields *β*-carboline through oxidation (Fig. 5) and the compound structures (Figs. 6, 7, 8, 9).

### Nitrogen-containing flavaglines

Flavaglines have garnered significant interest due to their ability to inhibit translation initiation by targeting eukaryotic translation initiation factor 4A (eIF4A). This mechanism has been shown to disrupt cancer cell proliferation and induce apoptosis, making flavaglines promising leads for anticancer drug development. Rocaglamide and its analogs have exhibited potent activity against hematologic malignancies, pancreatic cancer, and neuroblastoma in preclinical studies. Additionally, flavaglines demonstrate anti-inflammatory effects by modulating NF-κB signaling, a key pathway involved in immune responses and inflammation. Moreover, flavaglines such as aglaroxin derivatives have been investigated for their neuroprotective properties, showing potential in treating neurodegenerative diseases like Alzheimer’s and Parkinson’s. Some derivatives have also displayed antiviral activity, particularly against RNA viruses, highlighting their broad-spectrum therapeutic potential [98, 99].

**Fig. 10:**
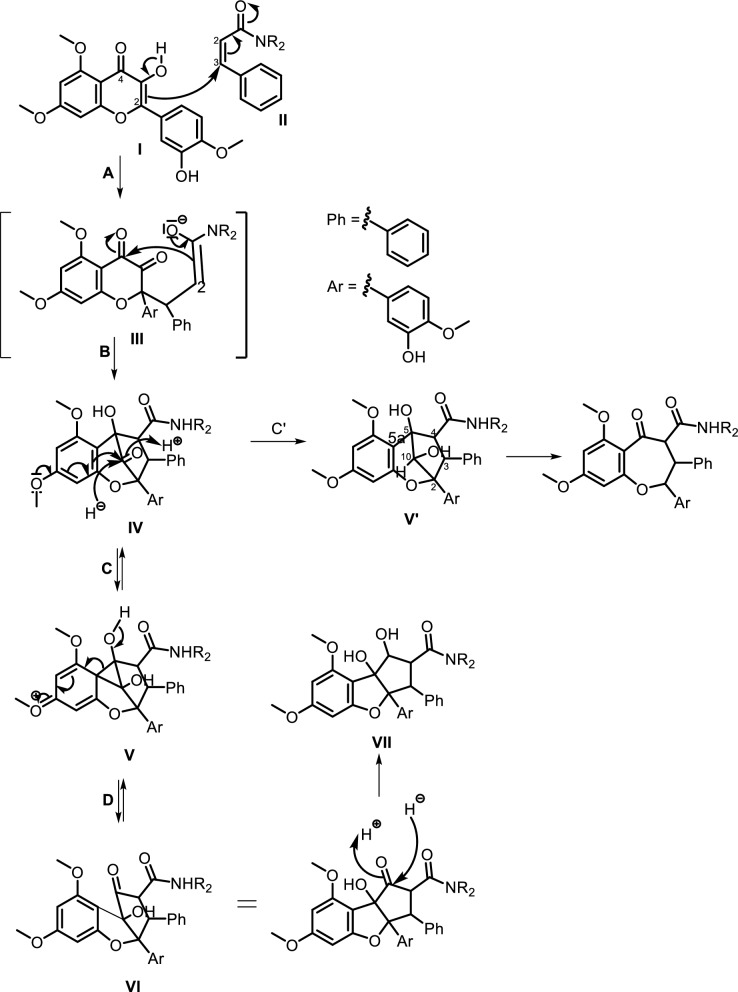
Proposed biosynthetic pathways of flavaglines

Despite their promising bioactivities, flavaglines face challenges related to bioavailability and synthetic accessibility. Ongoing research focuses on optimizing their pharmacokinetic properties and developing synthetic analogs with improved solubility and stability. High-throughput screening, computational modeling, and synthetic biology approaches are being employed to enhance flavagline-based drug discovery. Flavaglines have been identified as the only class of natural products peculiar to the genus Aglaia (Meliaceae). Due to their structural uniqueness and restricted taxonomic distribution, flavaglines are widely recognized as reliable chemical markers for species of this genus. The genus Aglaia remains an invaluable source of novel flavaglines, and further exploration of its chemical diversity is warranted. These nitrogen-bearing derivatives are relatively rare and exhibit significantly more complex molecular architectures, incorporating functionalities such as carboxamides, diamides, and nitrogen-containing heterocycles, which are uncommon in typical flavagline scaffolds. According to [100], the flavaglines can be divided into three subtypes namely, cyclopenta[b]benzofuran, cyclopenta[bc]benzopyran, and benzo[b]oxepines (see proposed biosynthetic pathway presented in Fig. 10). The enolate subunit I is believed to be incorporated into the α,β-unsaturated amide II through a Michael 1,4-type mechanism in the initial C,C linking step (step A), which occurred between C-2 of flavonoid I and C-3 of cinnamonate amide II. The preceding flavonoid’s C-4, which was a strongly active carbonyl group, could be attacked by the C-2 atom of the enolate III, creating a 5-membered ring that produced IV (step B). The formation of IV represented an essential metabolic step, serving as a precursor to derivatives of rocaglamide and aglain. Moreover, IV was a dehydroaglain derivative, and the equivalent aglain derivative V' (step C') could be obtained by a straightforward reduction step with H potentially representing NADPH or the associated nucleophile H. The intramolecular migration of electron-rich substituted aromatic rings of the phloroglucinol type was capable of being rearranged by migrating from the original C-4 to C-3 in the flavonoid. This reduction yielded derivative V to stabilize the strained molecule IV. The use of the cyclopropyl derivative V as an s-complex (steps C and D) allowed for a mechanistic understanding of this process as an electrophilic aromatic substitution. This process transformed the hydroxyketone IV into the isomeric hydroxyketone VI, which was a derivative of dehydrorocaglamide. A final stabilizing reduction (step E) yielded the derivative rocaglamide VII, confirming the potentially reversible mechanism [38, 101]. The cyclopenta[bc] benzopyran skeleton could be formally divided into the benzo[b]oxepine by oxidative cleavage at the methylene bridge connecting C-5 and C-10 [102].

Cyclorocaglamide (**114**) was isolated from the twigs of *Aglaia oligophylla* Miq. [103], while rocaglamide (**115**) has been isolated from *Aglaia elliptifolia* Merr. (syn. *Aglaia rimosa* (Blanco) Merr.), *Aglaia odorata* Lour. and *Aglaia formosana* Hayata (syn. *Aglaia elaeagnoidea* Benth.). Rocaglammides D (**116**), AB (**118**), I (**119**), Q (**121**), W (**122**), AY (**125**), and aglaroxin E (**117**) have also been successfully isolated from the twigs, flowers, bark, and roots of *Aglaia odorata* Lour.*, Aglaia duperreana* Pierre, and *Aglaia roxburghiana* Miq. [36–38, 103–107]. Structurally, most of these compounds preserve the cyclopenta[b]benzofuran or [bc]benzopyran core, but are further elaborated with nitrogen-containing moieties that often include amide linkages at C-2 or C-8b. These modifications result in increased molecular weight, additional hydrogen-bonding capacity, and altered polarity compared to their non-nitrogenous counterparts. Notable examples include 3'-hydroxy-8b-ethoxy-rocaglamide (**120**), 3'-hydroxy-8b-ethoxy-desmethylrocaglamide (**123**), aglaroxin A (**135**), and the highly modified compounds **150** and **151**, which feature multi-substituted diamide side chains fused to complex oxygenated skeletons. Compounds **120** and **123** were successfully purified by [106] from the flowers of Aglaia duperreana Pierre, in which the hydroxyl group at C-8b was substituted with an ethoxy group. Additionally, two known compounds have been isolated and elucidated, **130** and **131**. Didesmethylrocaglamide (**124**) was isolated from seeds of *Aglaia argentea* Blume [76] and leaves, twigs, and fruits of *Aglaia perviridis* Hiern [27]. A total of four rocaglamide derivatives were purified from *Aglaia odorata* Lour. twigs, where two new compounds were elucidated as 8b-methoxy-desmethylrocaglamide (**126**) and 3'-hydroxy-8b-methoxy-rocaglamide (**127**). Meanwhile, two known compounds were designated as 3'-hydroxy-desmethylrocaglamide (**128**), and 3'-hydroxydidesmethyl rocaglamide (**129**) [38, 107].

The compound 3'-methoxy-*N*-demethylrocaglamide (**132)** has been isolated from twigs and leaves of *Aglaia odorata* Lour. [35]*,* while **134** was obtained from roots and stems of *Aglaia elliptifolia* Merr. [108]. In addition to **117,** aglaroxin A (**135**) was found in *Aglaia roxburghiana* Miq.*, Aglaia oligophylla* Miq.*,* and *Aglaia edulis* A.Gray bark, including **136** and (**137**) [62]. Aglaroxin B (**138**), F (**139**), D (**140**), C (**146**), G (**147**), H (**148**), I (**149),** J (**169**), 14 (**170**), 15 (**171**), and 16 (**172**) were obtained from *Aglaia roxburghiana* Miq. stem barks [37]. Additionally*,* **140** was found in *Aglaia odorata* Lour. leaves [109] and *Aglaia duperreana* Pierre twigs [110]. Dehydroaglaiastatin (**141**) was identified as a compound present in the entire tree of the species *Aglaia odorata* Lour.*, Aglaia formosana* Hayata, and *Aglaia duperreana* Pierre*.* Aglaiformosanin (**142**), and a new cytotoxic cyclopenta[b]benzofuran derivative, was identified in the stem bark of *Aglaia formosana* Hayata. Furthermore, compound 3’-hydroxyaglaroxin C (**143**) was obtained from *Aglaia formosana* Hayata flowers and *Aglaia odorata* Lour. twigs and leaves [34, 105]. The roots of *Aglaia gracilis* A.C.Sm. contained new compounds namely, marikarin (**144**) and 3’-hydroxymarikarin (**145**). [111] reported the following new compounds, (1*R*,2*R*,3*S*,3a*R*,8b*S*,-1‴*S*,2‴*R*, 4‴*R*)-4‴-[(*R*)-1,2-dihydroxy ethyl]-1,8b-dihydroxy-8-methoxy-3a-(4-methoxy phenyl)-3-phenyl-1,2,3a,8b, 1‴,2‴,3‴,4‴-octa hydro-8H-cyclopenta [4, 5] furo[3,2-f] [1, 4] dioxino [2,3-b]benzo furan-2-carboxamide (**150)** and (1*R*,2*R*,3*S*,3a*R*,8b*S*)-4‴-{[(2‴*R*,4‴*R*)-4‴-[(*S*)-1,2-dihydroxyethyl]-3-hydroxy-1,4-dioxan-2-yl] oxy}-1,8b-dihydroxy-8-methoxy-3a-(4-methoxy phenyl)-3-phenyl-2,3,3a,8b-tetrahydro-1H-cyclopenta [b]benzofuran-2-carboxamide (**151),** which have been isolated from roots of *Aglaia perviridis* Hiern*.* Meanwhile, from the leaves of *Aglaia perviridis* Hiern and twigs of *Aglaia oligophylla* Miq*.*, ( ±) aglapernin (**152)** and isothapsakone A (**153**) have been isolated. The compound ponapensin (**154)** was reported by [112] in the stems and leaves of *Aglaia ponapensis* Kaneh.

This type of N-functionalization introduces significant conformational rigidity and new intermolecular interaction opportunities, especially through hydrogen bonding and dipole interactions, which are highly relevant in biological target binding. For example, in compound **129**, the nitrogen moiety contributes to enhanced cytotoxic activity through increased solubility and potential interaction with ribosomal eIF4A, a validated target of flavaglines. Similarly, compound **150**, which possesses an additional amide bridge and hydroxylated aliphatic side chain, may exhibit a unique three-dimensional pharmacophore capable of engaging in multivalent interactions with biological macromolecules. Interestingly, the presence of nitrogen at critical positions such as C-2 or C-8b also affects the electronic properties of the flavagline core, potentially modulating π-stacking and cation–π interactions involved in protein recognition. Furthermore, the substitution pattern, such as ethoxy groups (as in **120**) or fused 1,4-dioxino rings—could influence the molecule’s metabolic stability and resistance to enzymatic degradation, factors which are crucial for drug development [113]. succeeded in isolating eight derivatives of 2,3,4,5-tetrahydro-2,5-methanobenzo[b]oxepine, aglaodoratins A-G, and H (**189–195**, and **155**), and tetrahydrocyclopenta[b]benzofuran derivatives, aglaodoratin I (**133**), from the leaves of *Aglaia odorata* Lour. Research conducted by [114] also reported the existence of eight new rocaglate biosynthesis precursors. These included aglapervirisins B-G (**213–218**) and H-I (**156–157**), which are found in *Aglaia perviridis* Hiern leaves. Subsequently [26], reported that aglapervirisin J-M (**158–161**) had been discovered in the same species and plant parts.

The diversity in structural motifs among nitrogen-containing flavaglines reflects divergent biosynthetic strategies likely driven by ecological or evolutionary pressures. While many canonical flavaglines arise from polyketide–terpenoid hybridization followed by oxidative cyclization, these N-containing derivatives seem to follow more elaborate biosynthetic tailoring, possibly through non-ribosomal peptide synthetase (NRPS)-like enzymes or transaminase-mediated modifications. This hypothesis is supported by the observation that some of the most complex members (e.g., **150 and 151**) harbor multiple oxygen and nitrogen atoms within extended ring systems that cannot be easily rationalized by typical type I PKS/terpene biosynthesis alone. Further study showed that several new compounds, aglains A (**162**), B (**163**), and C (**164**) have been isolated from *Aglaia argentea* Blume leaves and possess a cyclopentatetrahydrobenzopyran type structure. *Aglaia forbesii* King barks and *Aglaia elliptica* Blume leaves yielded **162** [76, 78]. The leaves of *Aglaia laxiflora* Miq. yielded aglaxiflorin A (**165**), B (**166**), C (**182**), and D (**167**). Moreover, **167** was found in *Aglaia laxiflora* Miq., *Aglaia testicularis* C.Y.Wu leaves [73], *Aglaia odorata* Lour. leaves [34, 40], *Aglaia roxburghiana* Miq. stembarks [37], while **168** was purified from *Aglaia elliptifolia* Merr. leaves. [115] succeeded in isolating new flavagline compounds from *Aglaia edulis* (Roxb.) Wall. rootbarks, as derivatives of cyclopenta[bc]benzopyrans (**173–178**) and benzo[b]oxepines (**226–228**). Other new cyclopentatetrahydrobenzopyran compounds were obtained from *Aglaia forbesii* King bark, namely agla*forbesii*n A (**180**) and B (**181**) [76].

The genus *Aglaia* is very rich in flavagline compounds, containing diamide as a derivative. Moreover, two new diamide compounds have been discovered from the stems and leaves of *Aglaia elliptica* Blume*,* namely 10-*O*-acetylaglain B (**183**) and 4-epiaglain A (**184**) [78]. [67] explored *Aglaia gracilis* A.C.Sm. leaves and reported the presence of desacetylaglain. Aglain derivatives were isolated and elucidated with C-3'-hydroxyaglain C (**186**), C-19, C-3'-dihydroxyaglain C (**187**), and C-19-hydroxy, C-3'-methoxyaglain C (**188**) [38]. Recently, other compounds of aglains were found in *Aglaia odorata* Lour. twigs and leaves as well as agldorate A (**196**), B (**197**), and C (**198**) [34].

**Fig. 11:**
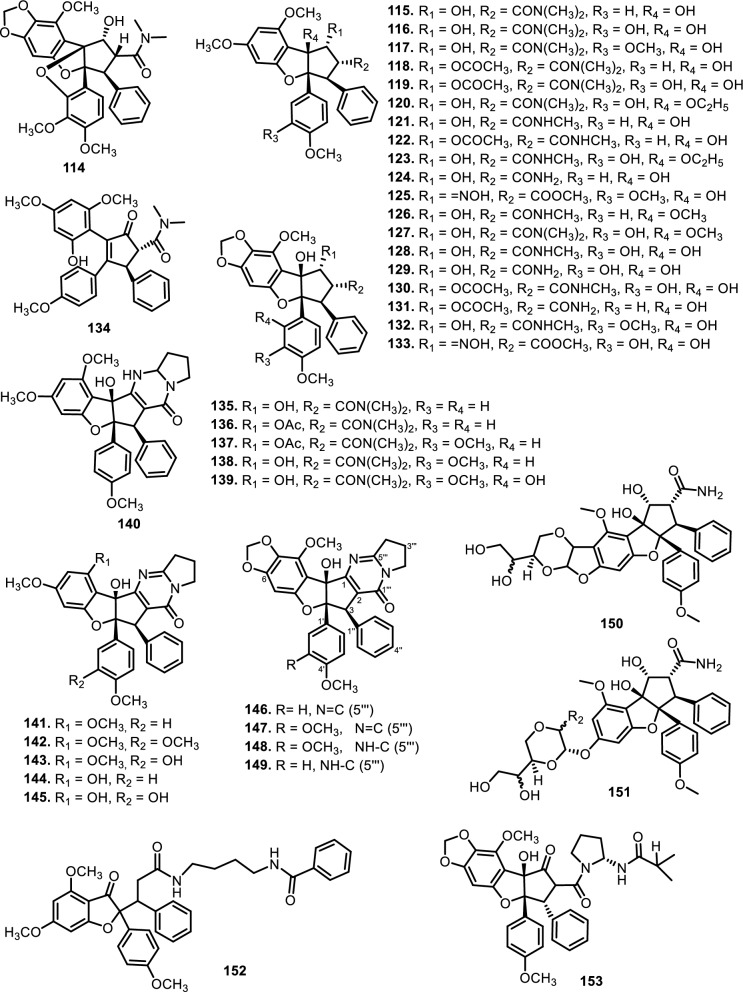
The chemical structures of compounds **114**–**153**

**Fig. 12:**
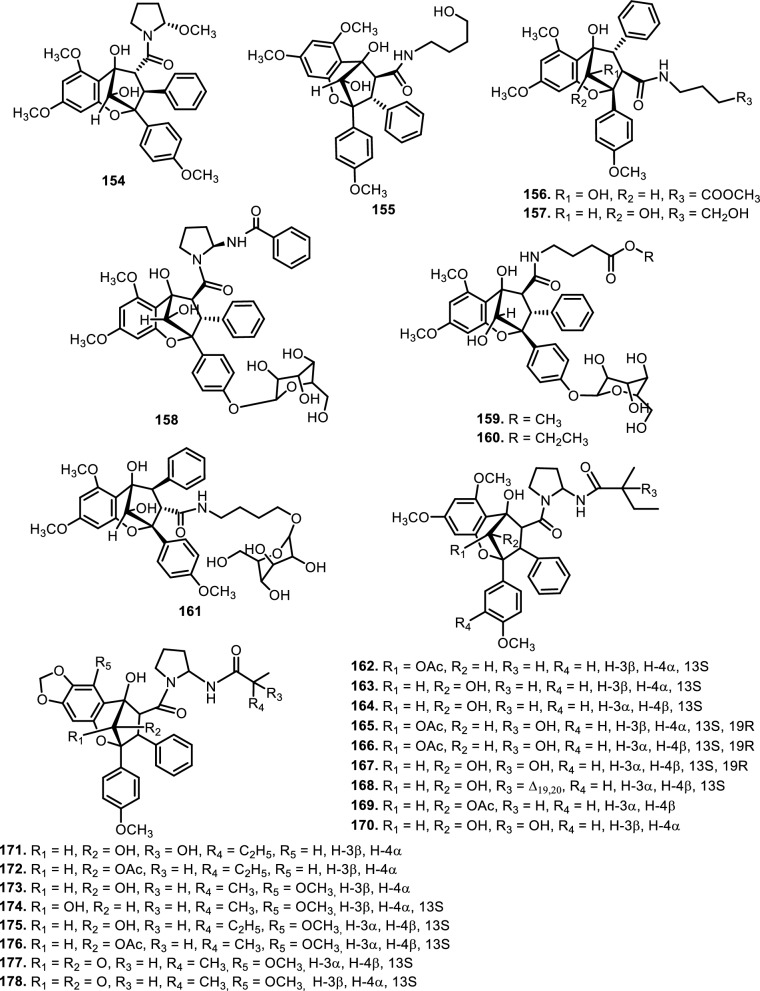
The chemical structures of compounds **154–178**

**Fig. 13:**
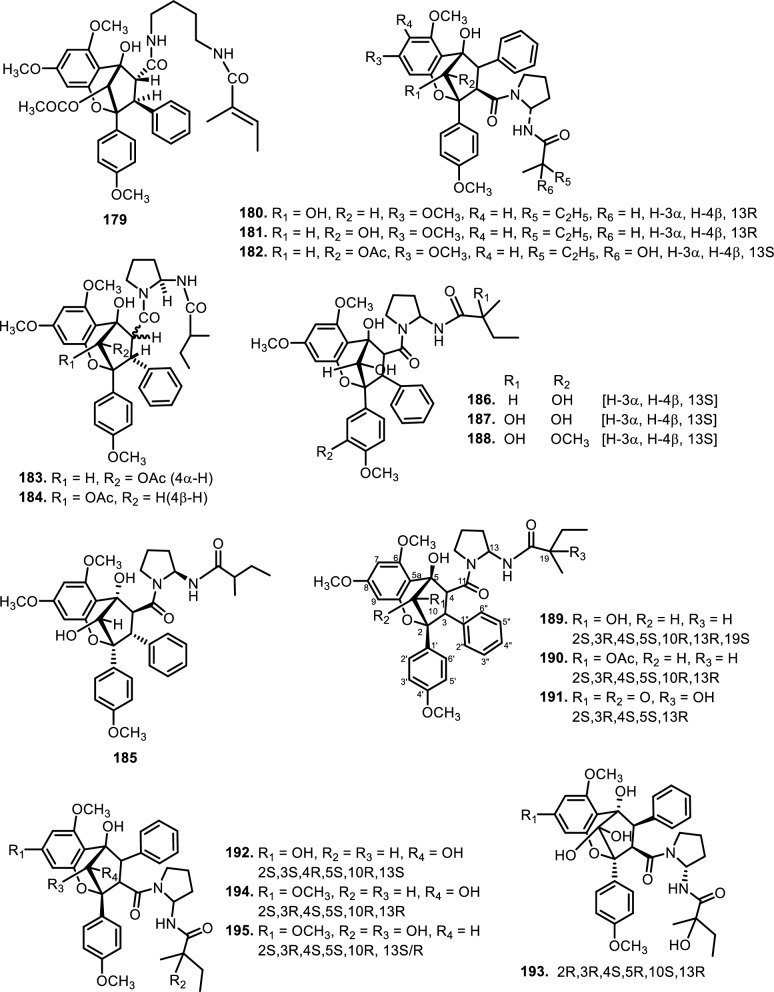
The chemical structures of compounds **179**–**195**

**Fig. 14:**
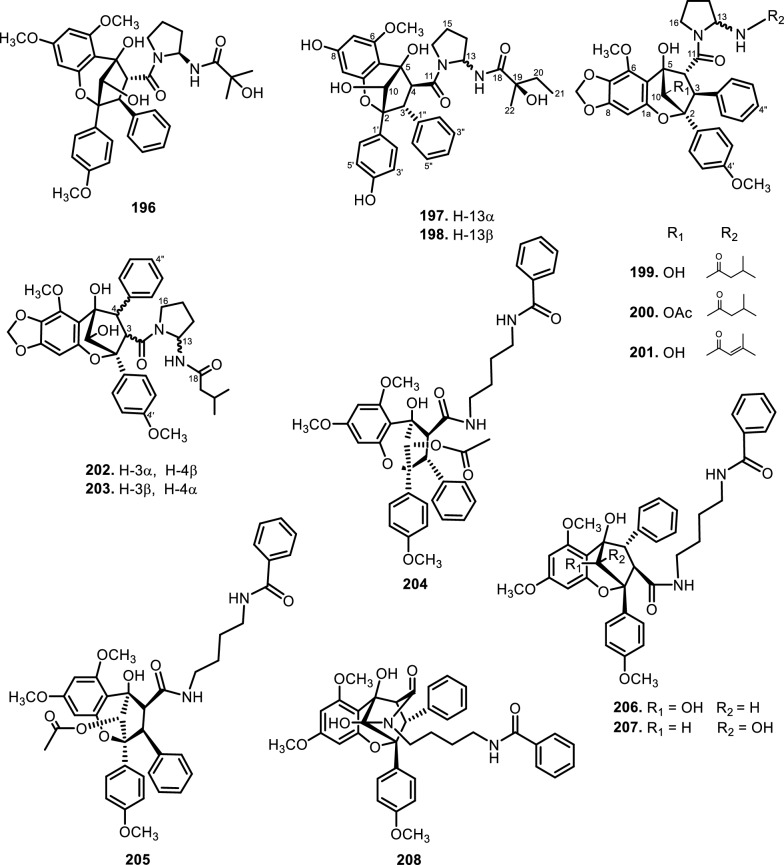
The chemical structures of compounds **196**–**208**

**Fig. 15:**
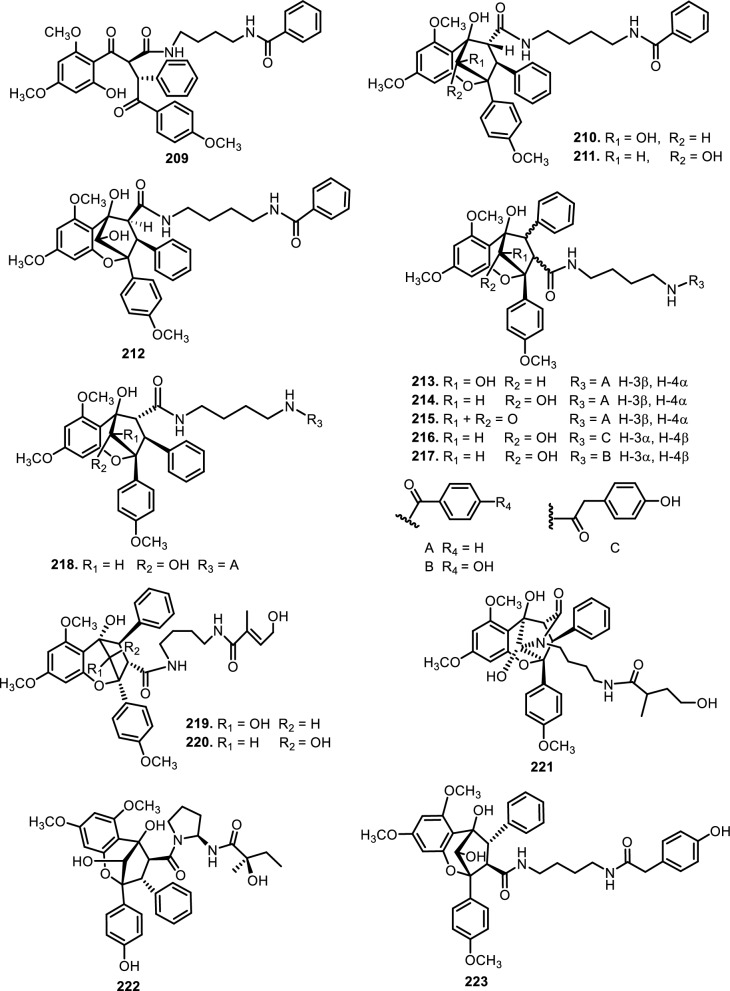
The chemical structures of compounds **209**–**223**

**Fig. 16:**
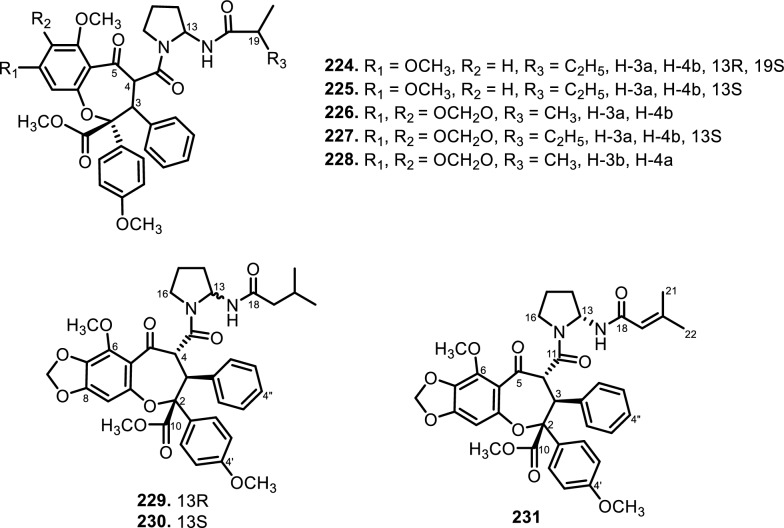
The chemical structures of compounds **224**–**231**

Cyclopenta[bc]benzopyrans are derivatives of flavagline, which is commonly found in *Aglaia*. A total of five new compounds have been found in the bark of *Aglaia edulis* (Roxb.) Wall., namely edulirin A (**199**), edulirin A-10-*O*-acetate (**200**), 19,20-dehydroedulirin A (**201**), isoedulirin A (**202**) and edulirin B (**203**) [62]. Two other compounds derived in the leaves of *Aglaia andamanica* Hiern, were pyramidaglain A (**204**) and B (**205**). The structure of compounds **204** and **205** is similar to foveoglin A (**206**), isolated from *Aglaia foveolata* Pannell leaves, with the exception of substituents placed at positions 3 and 4. Meanwhile, the C-10 epimer of compound **206** is referred to as foveoglin B (**207**). The presence of an acetyl group on C-10 led to isofoveoglin (**212**), which was also found in *Aglaia forbesii* King leaves. Compounds **208** and **209** are also new compounds from *Aglaia foveolata* Pannell leaves, with **209** having a cleaved oxepine ring. Compounds **210** and **211** were separated from the leaves of *Aglaia forbesii* King [66, 69]. From the leaves, twigs, and fruits of *Aglaia perviridis* Hiern derivatives of benzopyrans, perviridisin A (**219**) and B (**220**) were isolated. Interestingly, structurally distinct flavagline-related metabolites were also found in other species. Hydroxytigloyl-1,4-butanediamidecyclofoveoglin (**221)** was isolated from *Amoora cucullata* Roxb. leaves, 4′-O-demethyl-deacetylaglaxiflorin A (**222**) from *Aglaia odorata*, and Aglaiamide B (**223**) from *Aglaia perviridis* [27, 35, 64, 85] indicating convergent biosynthetic routes among Meliaceous genera. Additional benzooxepine derivatives have been discovered from *Aglaia* species, including forbaglin A (**224**) and B (**225**) from *A. forbesii* bark [76]. Likewise, edulisone A (**229**), edulisone B (**230**), and 19,20-dehydroedulisone A (**231**) were isolated from the bark of *A. edulis * (Table 2, Figs. 11, 12, 13, 14, 15, 16), expanding the chemical repertoire with oxygen-bridged ring systems that may contribute to their unique biological behavior. Taken together, these findings illustrate not only the rich chemodiversity of *Aglaia* but also highlight the frequent occurrence of structural motifs such as epimerization, acetylation, hydroxylation, and nitrogen incorporation. Such variations can profoundly influence the physicochemical and pharmacokinetic properties of these molecules, and continued phytochemical exploration of this genus holds promise for identifying novel bioactive compounds [62, 116].

**Table 2:** *N*-containing flavaglines on Meliaceae family

No	Compounds	Species	Plant part	Biological activity	References
Cyclopenta[b]benzofuran					
114	Cyclorocaglamide	*Aglaia oligophylla*	Twigs	*S. littoralis* (inactive up to 100 ppm)	[163]
115	Rocaglamide	*Aglaia elliptifolia*	Roots and stems	Not reported	[108]
			Stems	KB (IC_50_: 0.006 μg/mL); HCT-8 (IC_50_: 0.007 μg/mL); P-388 (IC_50_: 0.005 μg/mL); RPMI-7951 (IC_50_: 0.002 μg/mL); and TE-671 (IC_50_: 0.006 μg/mL)	[160]
		*Aglaia. odorata*	Leaves	*P. saucia* (EC_50_: 0.91 ppm; LD_50_: 0.28 ppm)	[36]
			Twigs	*S. litura* (LC_50_: 4.80 ppm)	[171]
			Roots	HL-60 (IC_50_: 0.007 μM); SMMC-7721 (IC_50_: 0.009 μM); MCF-7 (IC_50_: 0.008 μM); and SW480 (IC_50_: 0.031 μM)	[93]
		*Aglaia formosana*	Stem bark	HT-29 (ED_50_: 0.013 μg/mL)	[161]
116	3'-hydroxyrocaglamide (Rocaglamide D)	*A. odorata Aglaia duperreana*	Twigs Twigs	*S. littoralis* (LC_50_: 1.5 ± 0.65 ppm; EC_50_ = 0.21 ± 0.08 ppm)	[38] [110]
			Flowers	*S. littoralis* (LC_50_: 1.50 ppm; EC_50_ = 0.21 ppm)	[105]
			Roots	Not reported	[104]
117	Aglaroxin E	*A. odorata*	Twigs	*S. littoralis* (LC_50_: 1.0 ± 0.35 ppm; EC_50_: 0.09 ± 0.03 ppm)	[38]
		*A. duperreana*	Twigs	*S. littoralis* (LC_50_: 1.0 ± 0.35 ppm; EC_50_: 0.09 ± 0.03 ppm)	[110]
		*Aglaia roxburghiana*	Stem barks	*H. virescens*, *S. littoralis* and *P. xylostella* (12.5 mg/L giving 80–100% mortality) *D. balteata* (inactive)	[37]
118	Rocaglamide AB	*A. duperreana*	Bark	Not reported	[106]
			Roots	*S. littoralis* (LC_50_: 7.1 ppm and EC_50_: 0.43 ppm)	[104]
119	Rocaglamide I	*A. odorata*	Twigs	*S. littoralis* (LC_50_: 8.0 ± 1.44 ppm and EC_50_: 0.52 ± 0.08 ppm)	[38]
		*A. duperreana*	Flowers	Not reported	[105]
			Bark	Not reported	[106]
			Roots	Not reported	[104]
120	3'-hydroxy-8b-ethoxy-rocaglamide	*A. duperreana*	Flowers	*S. littoralis* (inactive)	[105]
121	Desmethylrocaglamide	*A. odorata*	Leaves	*P. saucia* (LD_50_ = 1.06 ppm; EC_50_ = 2.01 ppm)	[36]
			Twigs	K562 (IC_50_ = 9.5 μg/mL)	[107]
		*A. duperreana*	Roots	*S. littoralis* (LC_50_ = 1.3 ppm; EC_50_ = 0.27 ppm)	[104]
			Twigs	*S. littoralis* (LC_50_ = 1.3 ppm; EC_50_ = 0.27 ppm)	[110]
			Flowers	*S. littoralis* (LC_50_ = 1.3 ppm; EC_50_ = 0.27 ppm)	[105]
122	Rocaglamide W	*A. duperreana*	Bark	Not reported	[106]
			Roots	*S. littoralis* ((LC_50_ = 8.1 ppm; EC_50_ = 0.23 ppm)	[104]
			Flowers	Not reported	[105]
123	3'-hydroxy-8b-ethoxy-desmethylrocaglamide	*A. duperreana*	Flowers	*S. littoralis* (inactive)	[105]
124	Didesmethylrocaglamide	*Aglaia argantea*	Seeds	HT-29 cancer cell (ED_50_: 0.021 μM)	[76]
		*Aglaia perviridis*	Combination of the leaves, twigs, and fruits	CCD-112CoNl (ED_50_ > 50 μM) NF-κB (inactive, ED_50_ > 20 μM)	[76]
125	Rocaglamide AY	*A. oligophylla*	Leaves	Not reported	[106]
126	8b-methoxy-desmethylrocaglamide	*A. odorata*	Twigs	K562 (inactive)	[107]
127	3'-hydroxy-8b-methoxy-rocaglamide	*A. odorata*	Twigs	K562 (inactive)	[107]
128	3'-hydroxy-desmethylrocaglamide	*A. odorata*	Twigs	K562 (IC_50_: 4.5 μg/mL)	[107]
129	3'-hydroxydidesmethyl rocaglamide	*A. odorata*	Twigs	*S. littoralis* (LC_50_: 1.6 ± 0.55 ppm; EC_50_: 0.21 ± 0.07 ppm)	[38]
130	1-*O*-acetyl-3'-hydroxydesmethylrocaglamide	*A. duperreana*	Flowers	Not reported	[105]
131	1-*O*-acetyldidesmethylrocaglamide	*A. duperreana*	Flowers	*S. littoralis* (LC_50_ = 1.97 ppm; EC_50_ = 0.14 ppm)	[105]
132	3′-methoxy-*N*-demethylrocaglamide	*A. odorata*	Twigs and leaves	HeLa (IC_50_: 0.32 μM), SGC7901 (IC_50_: 0.12 μM), and A549 (IC_50_: 0.25 μM)	[35]
133	Aglaodoratin I	*A. odorata*	Leaves	Not reported	[113]
134	Dehydrorocaglamide	*A. elliptifolia*	Roots and stems	Not reported	[108]
135	Aglaroxin A	*Aglaia edulis*	Barks	Lu1 (ED_50_: 0.04 μg/mL), LNCaP (ED_50_: 0.02 μg/mL), MCF-7 (ED_50_: 0.06 μg/mL) and HUVEC (ED_50_: 0.1 μg/mL)	[62]
			Root barks	*S. littoralis* (LC_50_: 3.4 μg/g fr.wt; EC_50_: 0.21 μg/g fr.wt) respectively)	[37]
		*A. oligophylla*	Twigs	*S. littoralis* (LC_50_: 23.4 ppm; EC_50_: 0.49 ppm)	[164]
		*A. roxburghiana*	Stem barks	*H. virescens*, *P. xylostella*, and *D. balteata* (12.5, 3, and 3 mg/L giving 80–100% mortality,	[37]
136	Aglaroxin A 1-*O*-acetate	*A. edulis*	Barks	Lu1 (ED_50_: 0.001 μg/mL), LNCaP (ED_50_: 0.01 μg/mL), MCF-7 (ED_50_: 0.02 μg/mL) and HUVEC (ED_50_: 0.5 μg/mL)	[62]
137	3'-methoxyaglaroxin A 1-*O*-acetate	*A. edulis*	Barks	Lu1 (ED_50_: 0.5 μg/mL), LNCaP (ED_50_: 0.3 μg/mL), MCF-7 (ED_50_: 0.8 μg/mL) and HUVEC (ED_50_: 0.5 μg/mL)	[37]
138	Aglaroxin B	*A. roxburghiana*	Stem barks	*H. virescens*, *S. littoralis* and *P. xylostella* (50, 12.5, and 12.5 mg/L giving 80–100% mortality, respectively) *D. balteata* (inactive)	[37]
139	Aglaroxin F	*A. roxburghiana*	Stem barks	*H. virescens*, *S. littoralis*, *P. ylostella *and *D. balteata* (50, 50, 50, and 12.5 mg/L giving 80–100% mortality, respectively)	[37]
140	Aglaroxin D	*A. odorata*	Leaves	K-*ras*-NRK (IC_50_: 2.8 ng/mL), K-*ras*-NIH3T3 (IC_50_: 5.6 ng/mL), H-*ras*-NIH3T3 (IC_50_: 1.0 ng/mL), N-*ras*-NIH3T3 (IC_50_: 1.8 ng/mL), NRK (IC_50_: 2.1 ng/mL), and NIH3T3 (IC_50_: 4.1 ng/mL)	[109]
		*A. duperreana*	Twigs	*S. littoralis* (LC_50_: 1.1 ppm; EC_50_: 0.20 ppm)	[110]
		*A. roxburghiana*	Stem barks	*H. virescens*, *S. littoralis*, and *D. balteata*. (~ 100 mg/L giving 80–100% mortality)	[37]
		*A. gracilis*	Leaves	*S. littoralis* (LC_50_: 1.2 ppm; EC_50_: 0.09 ppm)	[67]
141	Dehydroaglaiastatin	*A. odorata*	Roots	Not reported	[172]
				K-*ras-*NRK (IC_50_: 81.0 ng/mL), K-*ras*-NIH3T3 (IC_50_: 8.9 ng/mL), H-*ras*-NIH3T3 (IC_50_: 7.8 ng/mL), N-*ras*-NIH3T3 (IC_50_: 8.0 ng/mL), NRK (IC_50_: 9.8 ng/mL), and NIH3T3 (IC_50_: 7.6 ng/mL)	[109]
			Leaves	HepG2 (IC_50_: 0.69 ± 0.06 μM)	[162]
			Whole tree	HEL (IC_50_: 0.03 ± 0.001 μM), and MDA-231 (IC_50_: 1.06 ± 0.27 μM)	[34]
			Twigs and leaves	A549 (ED_50_: 0.0012 μg/mL), HL-60 (ED_50_: 0.0010 μg/mL), HT-29 (ED_50_: 0.0015 μg/mL), KB (ED_50_: 0.01 μg/mL), and P-388 (ED_50_: 0.0018 μg/mL)	[161]
		*A. formosana*	Stem barks	*S. littoralis* (LC_50_: 1.6 ppm; EC_50_: 0.4 ppm)	[110]
		*A. duperreana*	Twigs	Not reported	[105]
			Flowers	Not reported	[105]
142	Aglaiformosanin	*A. formosana*	Stem barks	A549 (ED_50_: 0.014 μg/mL), HL-60 (ED_50_: 0.012 μg/mL), HT-29 (ED_50_: 0.011 μg/mL), KB (ED_50_: 0.025 μg/mL), and P-388 (ED_50_: 0.012 μg/mL)	[161]
143	3 ‘-hydroxyaglaroxin C	*A. duperreana*	Flowers	Not reported	[105]
		*A. odorata*	Twigs and leaves	HEL (IC_50_: 0.17 ± 0.06 μM) MDA-231 (IC_50_ > 20 μM)	[34]
144	Marikarin	*Aglaia gracilis*	Roots	*S. littoralis* (LC_50_: 12.2 (9.8–15.4) ppm; EC_50_: 0.69 (0.37–0.99) ppm)	[67]
145	3'-hydroxymarikarin	*A. gracilis*	Roots	Not reported	[67]
146	Aglaroxin C	*A. roxburghiana*	Stem barks	*S. littoralis* and *P. xylostella* (12.5 and 12.5 mg/L giving 80–100% mortality, respectively *H. virescens* and *D. balteata* (inactive)	[37]
147	Aglaroxin G	*A. roxburghiana*	Stem barks	*D. balteata* (50 mg/L giving 80–100% mortality *H. virescens, S. littoralis* and *P. xylostella* (inactive)	[37]
148	Aglaroxin H	*A. roxburghiana*	Stem barks	*P. xylostella* (100 mg/L giving 80–100% mortality *H. virescens*, *S. littoralis*, and *D. balteata* (inactive)	[37]
149	Aglaroxin I	*A. roxburghiana*	Stem barks	*D. balteata* (12.5 mg/L giving 80–100% mortality *H. virescens, S. littoralis* and *P. xylostella* (inactive)	[37]
150	(1*R*,2*R*,3*S*,3a*R*,8b*S*,-1‴*S*,2‴*R*, 4‴*R*)-4‴-[(*R*)-1,2-dihydroxy ethyl]-1,8b-dihydroxy-8-methoxy-3a-(4-methoxy phenyl)-3-phenyl-1,2,3a,8b, 1‴,2‴,3‴,4‴-octa hydro-8H-cyclopenta[4, 5] furo[3,2-f] [1, 4] dioxino [2,3-b]benzo furan-2-carboxamide	*A. perviridis*	Roots	HT-29 and PC-3 cancer cells (IC_50_ > 10 μM)	[111]
151	(1*R*,2*R*,3*S*,3a*R*,8b*S*)-4‴-{[(2‴*R*,4‴*R*)-4‴-[(*S*)-1,2-dihydroxyethyl]-3-hydroxy-1,4-dioxan-2-yl] oxy}-1,8b-dihydroxy-8-methoxy-3a-(4-methoxy phenyl)-3-phenyl-2,3,3a,8b-tetrahydro-1H-cyclopenta [b]benzofuran-2-carboxamide	*A. perviridis*	Roots	HT-29 and PC-3 (IC_50_: 2.3 μM for both)	[111]
152	( ±) aglapernin	*A. perviridis*	Leaves	*P. gingivalis* (MIC values: 125 μM)	[26]
153	Isothapsakone A	*A. oligophylla*	Twigs	*S. littoralis* (LC_50_: 6.52 ppm; EC_50_: 0.99 ppm)	[164]
Cyclopenta[bc]benzopyran					
154	Ponapensin	*Aglaia ponapensis*	Stems and leaves	NF-κB (IC_50_: 0.06 μM)	[112]
155	Aglaodoratin H	*A. odorata*	Leaves	Not reported	[113]
156	Aglapervirisin H	*A. perviridis*	Leaves	HepG2, HL-60, MCF-7, and HT-29 (inactive)	[114]
157	Aglapervirisin I	*A. perviridis*	Leaves	HepG2 (IC_50_: 27.7 μM), HL-60 (IC_50_: 29.4 μM), MCF-7 (IC_50_: 36.0 μM), and HT-29 (IC_50_: 23.1 μM)	[114]
158	Aglapervirisin J	*A. perviridis*	Leaves	Inhibitory in RAW 264.7 cell (IC_50_: 39.8 μM)	[26]
159	Aglapervirisin K	*A. perviridis*	Leaves	Inhibitory in RAW 264.7 cell (IC_50_: 40.7 μM)	[26]
160	Aglapervirisin L	*A. perviridis*	Leaves	Inhibitory in RAW 264.7 cell (IC_50_: 29.5 μM)	[26]
161	Aglapervirisin M	*A. perviridis*	Leaves	Inhibitory in RAW 264.7 cell (IC_50_: 43.7 μM)	[26]
162	Aglain A	*A. argantea*	Leaves	Not reported	[76]
		*Aglaia forbesii*	Barks	Not reported	[76]
		*Aglaia elliptica*	Leaves	Not reported	[78]
163	Aglain B	*A. argantea*	Leaves	*H. virescens*, *S. littoralis*, *P. xylostella*, and *D. balteata* (inactive)	[76]
164	Aglain C	*A. argantea*	Leaves	Not reported	[76]
165	Aglaxiflorin A	*Aglaia laxiflora*	Leaves	P-388 and MOLT-4 (inactive)	[77]
166	Aglaxiflorin B	*A. laxiflora*	Leaves	P-388 and MOLT-4 (inactive)	[77]
167	Aglaxiflorin D	*A. laxiflora*	Leaves	P-388 and MOLT-4 (inactive)	[77]
		*Aglaia testicularis*	Leaves	Not reported	[73]
		*A.odorata*	Leaves	Inhibitory in RAW 264.7 cells (IC_50_: 2.1 μM)	[40]
			Twigs and leaves	HEL (IC_50_: 6.25 ± 0.26 μM) and MDA-231 (IC_50_: 12.51 ± 0.31 μM)	[34]
		*A. roxburghiana*	Stem barks	*H. virescens*, *S. littoralis*, *P. xylostella*, and *D. balteata* (inactive)	[37]
168	Elliptifoline	*A. elliptifolia*	Leaves	A549 (ED_50_: 18.9 μg/mL) and P-388 (ED_50_: 3.41 μg/mL) HL-60, HT-29, and KB (inactive)	[72]
169	Aglaroxin J	*A. roxburghiana*	Stem barks	*H. virescens*, *S. littoralis*, *P. xylostella*, and *D. balteata* (inactive)	[37]
170	Aglaroxin 14	*A. roxburghiana*	Stem barks	*H. virescens*, *S. littoralis*, *P. xylostella*, and *D. balteata* (inactive)	[37]
171	Aglaroxin 15	*A. roxburghiana*	Stem barks	*H. virescens*, *S. littoralis*, *P. xylostella*, and *D. balteata* (inactive)	[37]
172	Aglaroxin 16	*A. roxburghiana*	Stem barks	*H. virescens*, *S. littoralis*, *P. xylostella*, and *D. balteata* (inactive)	[37]
173	Thapsakin B	*A. edulis*	Root barks	Not reported	[115]
174	Isothapsakin B	*A. edulis*	Root barks	Not reported	[115]
175	Homothapsakin A	*A. edulis*	Root barks	Not reported	[115]
		*A. oligophylla*	Twigs	*S. littoralis* (*inactive*)	[164]
176	Thapsakin A 10-*O*-acetate	*A. edulis*	Root barks	*S. littoralis* (inactive)	[115]
177	Thapsakon A	*A. edulis*	Root barks	Not reported	[115]
178	Thapsakon B	*A. edulis*	Root barks	Not reported	[115]
179	Grandiamide A	*Aglaia grandis*	Leaves	Not reported	[82]
180	Aglaforbesin A	*A. forbesii*	Barks	Not reported	[76]
181	Aglaforbesin B	*A. forbesii*	Barks	Not reported	[76]
182	Aglaxiflorin C	*A. laxiflora*	Leaves	P-388 and MOLT-4 (inactive)	[77]
183	10-*O*-acetylaglain B	*A. elliptica*	Leaves	Not reported	[78]
184	4-epiaglain A	*A. elliptica*	Leaves	Not reported	[78]
185	Desacetylaglain A	*A. gracilis*	Leaves	Not reported	[67]
186	C-3'-hydroxyaglain C	*A. odorata*	Leaves	Not reported	[38]
187	C-19, C-3'-dihydroxyaglain C	*A. odorata*	Leaves	Not reported	[38]
188	C-19-hydroxy, C-3'-methoxyaglain C	*A. odorata*	Leaves	Not reported	[38]
189	Aglaodoratin A	*A. odorata*	Leaves	MG-63, HT-29, and SMMC-7721 (IC_50_ > 10 μM)	[113]
190	Aglaodoratin B	*A. odorata*	Leaves	MG-63, HT-29, and SMMC-7721 (IC_50_ > 10 μM)	[113]
191	Aglaodoratin C	*A. odorata*	Leaves	MG-63 (IC_50_: 1.2 μM) and HT-29 (IC_50_: 0.097 μM) SMMC-7721 (IC_50_ > 10 μM)	[113]
192	Aglaodoratin D	*A. odorata*	Leaves	MG-63 cancer cell (IC_50_: 0.75 μM) HT-29 and SMMC-7721 (IC_50_ > 10 μM)	[113]
193	Aglaodoratin E	*A. odorata*	Leaves	SMMC-7721 cancer cell (IC_50_: 6.25 μM) MG-63 and HT-29 (IC_50_ > 10 μM)	[113]
194	Aglaodoratin F	*A. odorata*	Leaves	MG-63, HT-29, and SMMC-7721 (IC_50_ > 10 μM)	[113]
195	Aglaodoratin G	*A. odorata*	Leaves	Not reported	[113]
196	Agldorate A	*A. odorata*	Twigs and leaves	HEL (IC_50_: 8.40 ± 0.85 μM) MDA-231cell (IC_50_ > 20 μM)	[34]
197	Agldorate B	*A. odorata*	Twigs and leaves	HEL and MDA-231 cells (IC_50_ > 20 μM)	[34]
198	Agldorate C	*A. odorata*	Twigs and leaves	HEL and MDA-231 cells (IC_50_ > 20 μM)	[34]
199	Edulirin A	*A. edulis*	Barks	Lu1, LNCaP, MCF-7 and HUVEC (ED_50_ > 5 μg/mL)	[62]
200	Edulirin A 10-*O*-acetate	*A. edulis*	Barks	Lu1, LNCaP, MCF-7 and HUVEC (ED_50_ > 5 μg/mL)	[62]
201	19,20-dehydroedulirin A	*A. edulis*	Barks	Lu1, LNCaP, MCF-7 and HUVEC (ED_50_ > 5 μg/mL)	[62]
202	Isoedulirin A	*A. edulis*	Barks	Lu1, LNCaP, MCF-7 and HUVEC (ED_50_ > 5 μg/mL)	[62]
203	Edulirin B	*A. edulis*	Barks	Lu1, LNCaP, MCF-7 and HUVEC (ED_50_ > 5 μg/mL)	[62]
204	Pyramidaglain A	*Aglaia andamanica*	Leaves	Not reported	[68]
205	Pyramidaglain B	*A. andamanica*	Leaves	Not reported	[68]
206	Foveoglin A	*Aglaia foveolata*	Leaves	Lu1 (ED_50_: 1.8 μM), LNCaP (ED_50_: 1.4 μM), and MCF-7 (ED_50_: 1.8 μM)	[66]
207	Foveoglin B	*A. foveolata*	Leaves	Lu1, LNCaP, and MCF-7 (ED_50_ > 20 μM)	[66]
208	Cyclofoveoglin	*A. foveolata*	Leaves	Lu1 (ED_50_: 18.1 μM), LNCaP (ED_50_: 16.0 μM), and MCF-7 (ED_50_: 13.5 μM)	[66]
209	Secofoveoglin	*A. foveolata*	Leaves	Lu1, LNCaP, and MCF-7 (ED_50_ > 20 μM)	[66]
210	Des-acetylpyramidaglain A	*A. forbesii*	Leaves	Not reported	[69]
211	Des-acetylpyramidaglain C	*A. forbesii*	Leaves	Not reported	[69]
212	Isofoveoglin	*A. foveolata*	Leaves	Lu1 (ED_50_: 17.5 μM), LNCaP (ED_50_: 21 μM), and MCF-7 (ED_50_: 16.1 μM)	[66]
		*A. forbesii*	Leaves	*M. tuberculosis* H37Ra (MIC-value at 25 μg/mL) *H. simplex* (moderately)	[69]
213	Aglapervirisin B	*A. perviridis*	Leaves	HepG2, HL-60, MCF-7, and HT-29 cells (IC_50_ > 50 μM)	[114]
214	Aglapervirisin C	*A. perviridis*	Leaves	HepG2, HL-60, MCF-7, and HT-29 cells (IC_50_ > 50 μM)	[114]
215	Aglapervirisin D	*A. perviridis*	Leaves	HepG2, HL-60, MCF-7, and HT-29 cells (IC_50_ > 50 μM)	[114]
216	Aglapervirisin E	*A. perviridis*	Leaves	HepG2 (IC_50_: 10.9 μM), HL-60 (IC_50_: 2.2 μM), MCF-7 (IC_50_: 8.5 μM). and HT-29 (IC_50_: 1.4 μM)	[114]
217	Aglapervirisin F	*A. perviridis*	Leaves	Not reported	[114]
218	Aglapervirisin G	*A. perviridis*	Leaves	HepG2, HL-60, MCF-7, and HT-29 cells (IC_50_ > 50 μM)	[114]
219	*Perviridis*in A	*A. perviridis*	Combination of the leaves, twigs, and fruits	HT-29 cancer cell (ED_50_ > 10 μM)	[27]
220	Perviridisin B	*A. perviridis*	Combination of the leaves, twigs, and fruits	HT-29 (ED_50_: 0.46 μM) CCD-112CoNl (ED_50_ > 50 μM) NF-κB (ED_50_: 2.4 μM)	[27]
221	Hydroxytigloyl-1,4-butanediamidecyclofoveoglin	*Amoora cucullata*	Leaves	TRAIL resistance-overcoming activity (strong)	[85]
222	4'-*O*-demethyl-deacetylaglaxiflorin A	*A. odorata*	Twigs and leaves	HeLa, SGC7901, and A549 (inactive)	[35]
223	*Aglaia*mide B	*A. perviridis*	Leaves	Not reported	[64]
Benzo[b]oxepines					
224	Forbaglin A	*A. forbesii*	Barks	Not reported	[96]
225	Forbaglin B	*A. forbesii*	Root barks	*S. littoralis* (inactive)	[97]
226	Thapoxepine A	*A. forbesii*	Barks	Not reported	[97]
227	Homothapoxepine A	*A. edulis*	Root barks	Not reported	[115]
228	Thapoxepine B	*A. edulis*	Root barks	Not reported	[115]
229	Edulisone A	*A. edulis*	Barks	Not reported	[116]
230	Edulisone B	*A. edulis*	Barks	Not reported	[116]
231	19,20-dehydroedulisone A	*A. edulis*	Barks	Lu1, LNCaP, MCF-7 and HUVEC (ED_50_ > 5 μg/mL)	[62]

### Nitrogen-containing limonoids

**Fig. 17:**
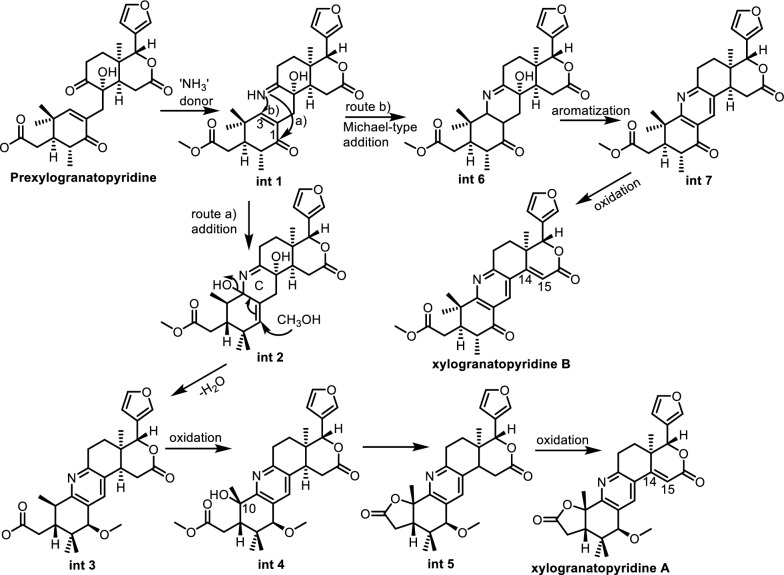
Proposed biosynthetic pathway to nitrogen-containing limonoids

Limonoids, a major class of highly oxygenated triterpenoids in the Meliaceae family, are typically rich in oxygen atoms. However, the rare occurrence of nitrogen-containing limonoids represents a significant structural deviation, introducing unique features that often correlate with enhanced or diversified biological activities. This uncommon modification has drawn considerable interest for its potential in natural product chemistry and drug development. Figure 17 presents the proposed biosynthetic pathway leading to nitrogen-containing limonoids, using xylogranatopyridines A and B as examples. Prexylogranatopyridine is proposed as a common biosynthetic precursor for both xylogranatopyridines A and B. This compound may undergo a formal Schiff base reaction with ammonia to yield the key intermediate, int 1, which can proceed via two alternative pathways: a nucleophilic addition at the C-1 position (route a) forming int 2, or an intramolecular Michael addition at C-3 (route b) leading to int 6. In route a, int 2 may then undergo aromatization of ring C to form a pyridine ring, followed by oxidation at C-10 and γ-lactonization, ultimately resulting in the formation of the Δ^14(15)^ double bond and production of xylogranatopyridine A. In route b, aromatization of int 6 could yield intermediate int 7, which upon oxidation may lead to the formation of xylogranatopyridine B [117].

Among Meliaceae genera, *Xylocarpus* is notably prolific in nitrogenous limonoids, with granatoine (**232**) [118], from the seeds, twigs, and leaves of *Xylocarpus granatum* J.Koenig*,* xylogranatine F (**234**), xylogranatopyridines A (**237**) and B (**238**) [117, 119]. Meanwhile, hainangranatumin G (**233**), xylogranatine G (**235**), H (**236**), and xylomexicanin E (**239**) were only found in the seeds of *Xylocarpus granatum* J.Koenig [119–121]. Compounds **232–238** are aromatic B-ring limonoids that feature a pyridine ring with variations in the substitutions at the C-3 position. Compound **232** contains a hydroxyl group at the β-position, while compound **236** has a hydroxyl group at the α-position. Compound **233** is substituted with an ethoxy group, **234** has a hydrogen atom, and **235** features an acetyl group. Compound **237** contains a methoxy group, while **238** is characterized by a carbonyl group. Thaixylomolin B (**240**) and C (**241**) were isolated for the first time from seeds of *Xylocarpus moluccensis* M.Roem. that contain a unique pentasubstituted pyridine scaffold [122].

Beyond *Xylocarpus*, nitrogen-containing limonoids have been identified in *Azadirachta indica* A. Juss. seeds, including azadiramide A (**242**) and B (**243**) [123, 124]. *Azadirachta indica* A. Juss. leaves were found to contain compounds **244** and **247** [125, 126], while Salannolactam-(21) (**245)** and Salannolactam-(23) (**246)** have been obtained from *Azadirachta indica* A. Juss. seed kernels [127]. Another Meliaceae genus that yielded nitrogen-containing limonoids was *Entandrophragma.* From *Entandrophragma angolense* C.DC. stem barks, the compound entangolensin K (**248**) was isolated was isolated [128]. Entanutilin B (**249**), a mexicanolide-type limonoid with 1,8-ketals was obtained from the stem bark of *Entandrophragma utile* Sprague. [129, 130] also isolated **249** with other new limonoid compounds of the highly oxygenated and rearranged phragmalin type. These included entanutilin C (**250**), entanutilin J (**251**), 12α-acetoxyphragmalin-3 nicotinate-30-isobutyrate (14) (**252**), entanutilin P (**253**), and entanutilin Q (**254**). Compounds **250**, **252**, and **253** have a characteristic orthoester bridge spanning C-1, C-8, and C-9. These compounds exhibit extensive structural modifications, including multiple nicotinyl ester, isobutyryl, and acetyl groups at C-3, C-12, and C-30, respectively. A fused δ-lactone ring at C-19 and the presence of a substituted furan moiety further distinguish these molecules).

Extensive structural diversity has also been reported from *Amoora tsangii* (Merr.) X.M.Chen, which yielded twelve nitrogenous limonoids, amooramides A–L (**255–266**). These compounds are characterized by a highly oxygenated tetranortriterpenoid skeleton bearing a fused ε-lactone ring and diverse lactam side chains at C-17. Compounds **255–266** exhibit structural diversity, primarily variations including acetoxy, formyloxy, benzoyloxy, acyloxy, and 2-methylbutanoyloxy group at C-11 and C-12, and different acyl or alkyl groups attached to the lactam side chain at C-17 [131]. Similarly, *Munronia* species such as *Munronia henryi* Harms and *Munronia pinnata* (Wall.) W.Theob. yielded munroniamide (**267**), munronin D (**268**), and prieurianin-type limonoids muropin A (**269**) and B (**270**) characterized by α,β-unsaturated γ-lactam motifs [132, 133]*.* Subsequently, it was revealed the *Turraea* genus produced nitrogen-containing limonoids, namely turraparvin D (**271**) from *Turraea parvifolia* Deflers. seeds and turrapubesin B (**272**) from *Turraea pubescens* Hell. twigs and leaves [134–136].

Further investigations into Meliaceae alkaloids have highlighted the presence of azadirone- and gedunin-type limonoids. Toonasinemine A–G (**273–279**), five of which contain modified furan ring (**275–279*)***, were isolated from the root bark of *Toona sinensis* (A.Juss.) M.Roem. [137]*.* Other limonoids with a rare lactam E ring, toonasins A–C (**280–282**), were identified in the bark of the same species [138]. Meanwhile, [139] examined *Toona ciliata* M.Roem. twigs and observed that toonaolide I (**283**), R (**284**), X (**285**) in 2020, toonaone I (**290**), and ciliatones C-F (**291–294**) in 2021 were present [140]. Compounds **291–294** exhibit variability at positions C-4 and C-6, with substituents including methyl, acetoxy, and formyl groups. Specifically, ciliatone C (**291**) bears a methyl group at C-4 and no substitution at C-6, while ciliatone D (**292**) features an additional acetoxy group at C-6. Ciliatone E (**293**) presents a formyl group at C-4 and an acetoxy group at C-6, and ciliatone F (**294**) lacks substitution at C-4 but contains an acetoxy group at C-6. Complementing this, Toononoid B, D, H, and Toonanoronoid H (**286–289**) were isolated from leaves and twigs *Toona ciliata var. yunnanensis* [141].

**Fig. 18:**
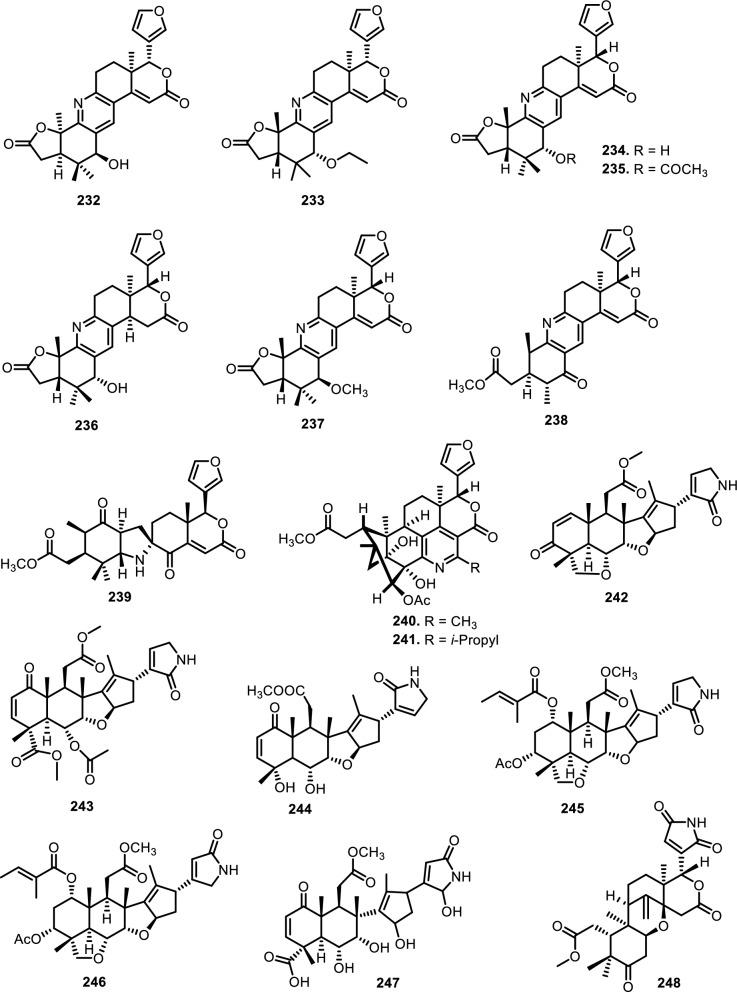
The chemical structures of compounds **232**–**248**

**Fig. 19:**
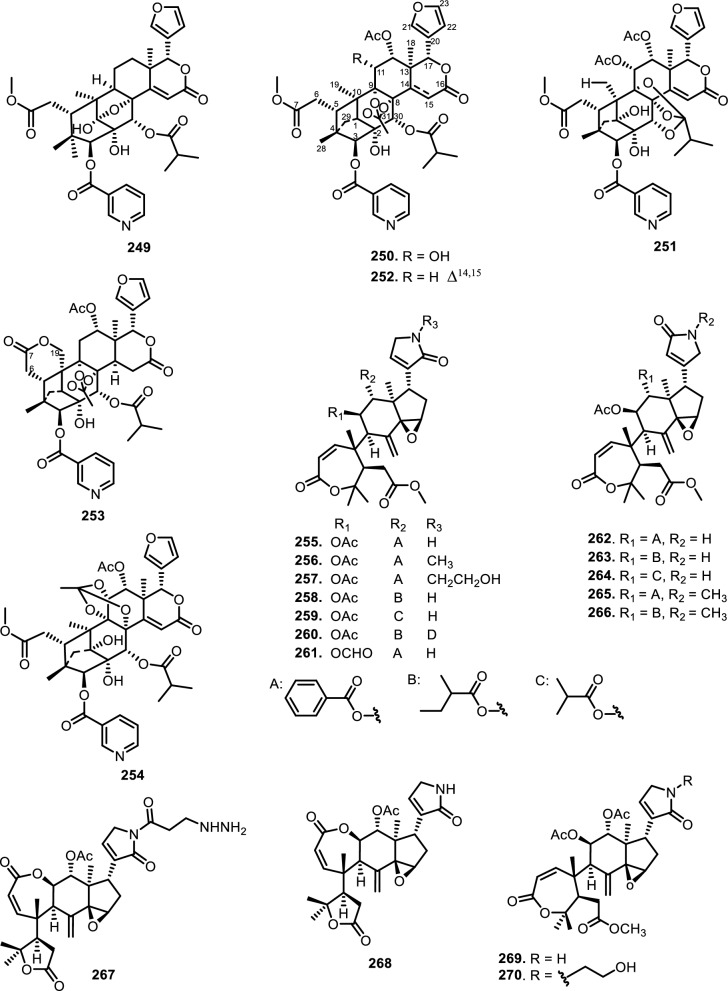
The chemical structures of compounds **249–270**

**Fig. 20:**
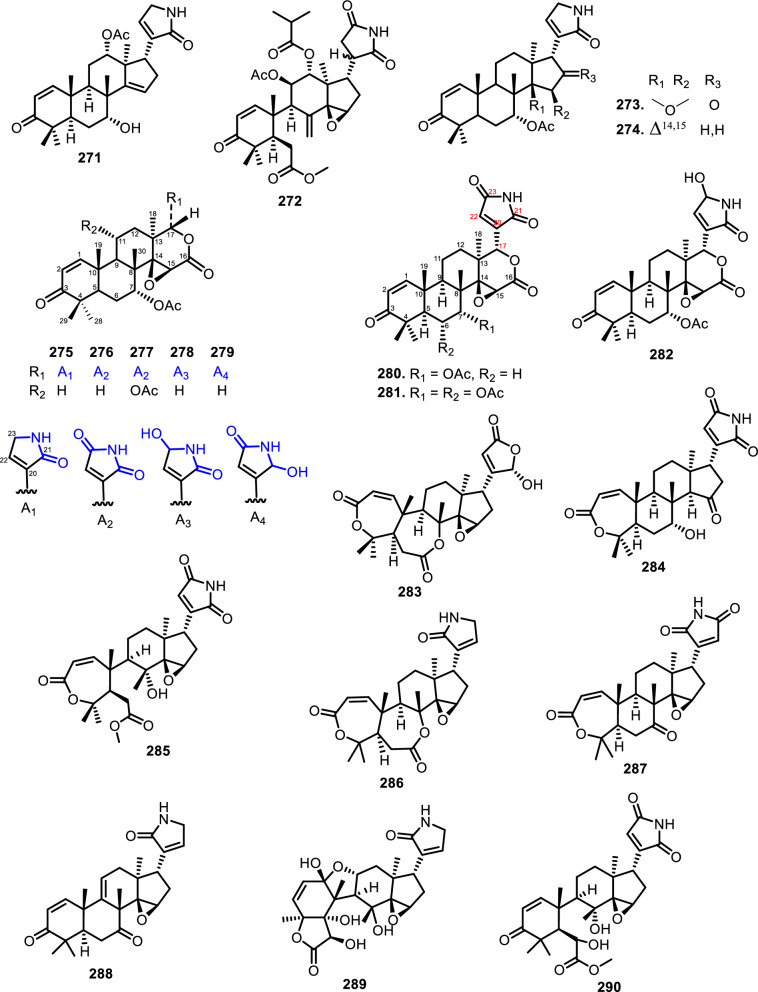
The chemical structures of compounds **271**–**290**

**Fig. 21:**
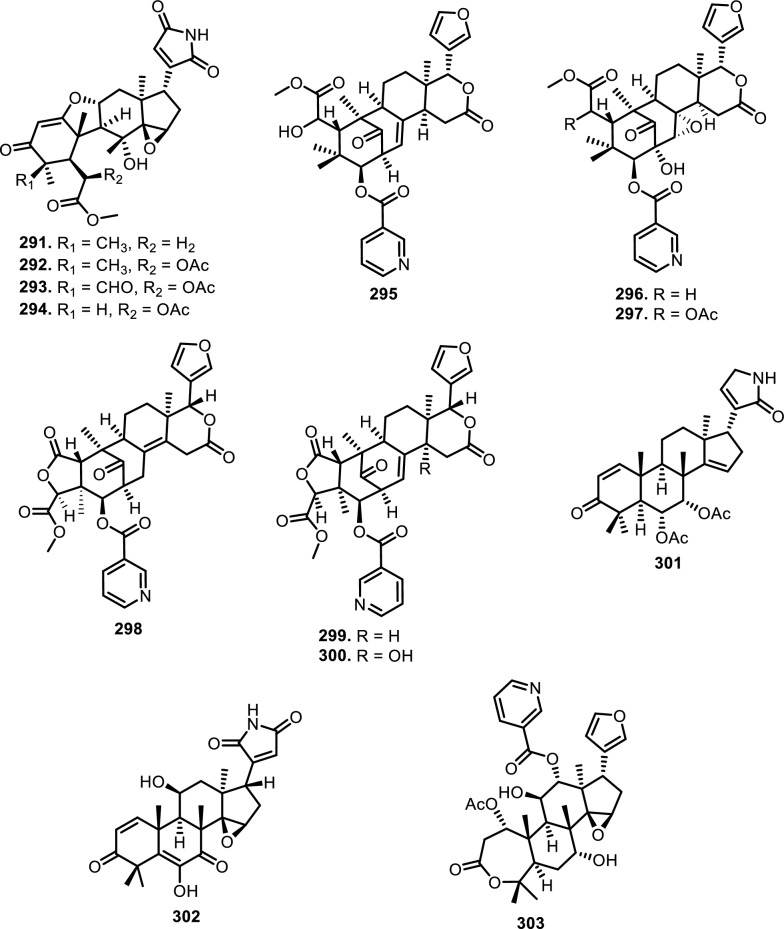
The chemical structures of compounds **291**–**303**

Compared to *Toona sinensis* (A.Juss.) M.Roem., *Trichilia sinensis* Bentv. was reported to produce new compounds namely trichinenlide A (**295**), F (**296**), G (**297**), and trichiliasinenoid D (**298**), from leaves and twigs [142, 143]. Meanwhile, *Trichilia connaroides* (Wight & Arn.) Bentv. from the leaves and twigs were discovered to make triconoid A (**299**) and B (**300**) [142–144]. These compounds are limonoid derivatives featuring a mexicanolide skeleton, which have β-substituted furan ring and multiple ester substitutions. Notably, **297** contains an additional acetoxy substituent compared to **296**, while **300** differs from **299** by the presence of a hydroxyl group at a previously unsubstituted position. Other nitrogen-containing limonoid derivatives included 6α,7α-diacetoxy-3-oxo-24,25,26,27-tetranorapotirucalla-1,14,20(22)-trien-21,23-lactam (**301**) from *Chisocheton paniculatus* Hiernfruit, walsunoid I (**302**) from *Walsura Robusta* Roxb.leaves, and aphanalide M (**303**) in *Aphanamixis grandifolia* Blume fruit [145–147], further underscoring the structural breadth and ecological significance of nitrogenous limonoids in the Meliaceae family (Table 3, Figs. 18, 19, 20, 21).

**Table 3:** *N*-containing limonoids on Meliaceae family

No	Compounds	Species	Plant part	Biological activity	References
232	Granatoine	*Xylocarpus granatum*	Fruits	Not reported	[118]
233	Hainangranatumin G	*X. granatum*	Seeds	Not reported	[120]
234	Xylogranatin F	*X. granatum*	Seeds	*M. separata* (antifeedant rates at 24, 48, and 72 h: 50.0, 55.2, 57.7%, respectively)	[119]
			Twigs and leaves	Not reported	[117]
235	Xylogranatin G	*X. granatum*	Seeds	*M. separata* (AFC_50_ at 24 and 48 h = 0.31 and 0.30 mg/mL, respectively	[119]
236	Xylogranatin H	*X. granatum*	Seeds	Not reported	[119]
237	Xylogranatopyridine A	*X. granatum*	Twigs and leaves	PTP1B (IC_50_: 22.9 μM)	[117]
238	Xylogranatopyridine B	*X. granatum*	Twigs and leaves	PTP1B (inactive)	[117]
239	Xylomexicanin E	*X. granatum*	Seeds	A549, RERF, PC-3, PC-6. QG-56, and QG-90 (IC_50_ > 100 μM)	[121]
240	Thaixylomolin B	*Xylocarpus moluccensis*	Seeds	Inhibitory in RAW 264.7 cell (IC_50_: 84.3 μM)	[122]
241	Thaixylomolin C	*X. moluccensis*	Seeds	Not reported	[122]
242	Azadiramide A	*Azadirachta indica*	Seeds	MDA-MB-231 (IC_50_: 2.70 ± 0.63 μM)	[123]
243	Azadiramide B	*A. indica*	Seeds	MDA-MB-231 (IC_50_ = 15.73 ± 6.07 μM)	[124]
244	Nimbandiolactam-21	*A. indica*	Leaves	α-glucosidase inhibitory activity (IC_50_: 79.7 μM)	[125]
245	Salannolactam-(21)	*A. indica*	Seed kernels	*E. varivestis* (strong)	[127]
246	Salannolactam-(23)	*A. indica*	Seed kernels	*E. varivestis* (strong)	[127]
247	Nimbic acid B	*A. indica*	Leaves	Allelopatic activity (IC_50_: 5.7–210 μM)	[126]
248	Entangolensin K	*Entandrophragma angolense*	Stem barks	HepG2 and MCF-7 (IC_50_ > 50 μM) inhibitory in RAW 264.7 cell (IC_50_: 7.94 ± 1.53 μM)	[128]
249	Entanutilin B	*Entandrophragma utile*	Stem barks	Inhibitory in RAW 264.7 cell (inactive)m MCF-7/DOX cells (inactive)	[173]
250	Entanutilin C	*E. utile*	Stem barks	MCF-7/DOX cells (inactive)	[129]
251	Entanutilin J	*E. utile*	Stem barks	MCF-7/DOX cells (inactive)	[129]
252	12α-acetoxyphragmalin-3 nicotinate-30-isobutyrate (14)	*E. utile*	Stem barks	MCF-7/DOX cells (inactive)	[129]
253	Entanutilin P	*E. utile*	Stem barks	MCF-7/DOX cells (inactive) inhibitory in RAW 264.7 cell (inactive)	[130]
254	Entanutilin Q	*E. utile*	Stem barks	MCF-7/DOX cells (inactive) inhibitory in RAW 264.7 cell (inactive)	[130]
255	Amooramide A	*Amoora tsangii*	Twigs and leaves	TNF-α induced κB activity (inactive) HepG2 (inactive at 10 μM)	[131]
256	Amooramide B	*A. tsangii*	Twigs and leaves	NF-κB (inactive) HepG2 cell line (inactive at 10 μM)	[131]
257	Amooramide C	*A. tsangii*	Twigs and leaves	Not reported	[131]
258	Amooramide D	*A. tsangii*	Twigs and leaves	NF-κB (inactive) HepG2 cell line (inactive at 10 μM)	[131]
259	Amooramide E	*A. tsangii*	Twigs and leaves	Not reported	[131]
260	Amooramide F	*A. tsangii*	Twigs and leaves	Not reported	[131]
261	Amooramide G	*A. tsangii*	Twigs and leaves	Not reported	[131]
262	Amooramide H	*A. tsangii*	Twigs and leaves	NF-κB (inactive) HepG2 cell line (inactive at 10 μM)	[131]
263	Amooramide I	*A. tsangii*	Twigs and leaves	NF-κB (64% at 10 μM) HepG2 cell line (inactive at 10 μM)	[131]
264	Amooramide J	*A. tsangii*	Twigs and leaves	Not reported	[131]
265	Amooramide K	*Amoora tsangii*	Twigs and leaves	Not reported	[131]
266	Amooramide L	*A. tsangii*	Twigs and leaves	Not reported	[131]
267	Munroniamide	*Munronia henryi*	Whole bodies	*P. brassicae* L (AR: 27.6%)	[132]
268	Munronin D	*M. henryi*	Whole bodies	*P. brassicae* L (AR: 28.0%)	[133]
269	Munropin A	*Munronia pinnata*	Aerial parts	Hela and A549 (inactive)	[134]
270	Munropin B	*M. pinnata*	Aerial parts	Hela and A549 (inactive at 100 μM)	[135]
271	Turraparvin D	*Turraea parvifolia*	Seeds	Not reported	[136]
272	Turrapubesin B	*Turraea pubescens*	Twigs and leaves	P-388 and A549 (inactive)	[137]
273	Toonasinemine A	*Toona sinensis*	Root barks	HepG-2 (IC_50_: 40.67 ± 3.23 μM) MCF-7 and U2OS (IC_50_ > 50 μM) inhibitory in RAW 264.7 cell (IC_50_: 10.21 ± 2.34 μM)	[137]
274	Toonasinemine B	*T. sinensis*	Root barks	HepG-2, MCF-7 and U2OS (IC_50_ > 50 μM inhibitory in RAW 264.7 cell (IC_50_: 20.05 ± 1.68 μM)	[137]
275	Toonasinemine C	*T. sinensis*	Root barks	HepG-2, MCF-7, U2OS, and RAW 264.7 (IC_50_ > 50 μM)	[137]
276	Toonasinemine D	*T. sinensis*	Root barks	HepG-2 (IC_50_: 11.63 ± 1.02 μM) and MCF-7 (IC_50_: 36.77 ± 2.79 μM) U2OS and RAW 264.7 (IC_50_ > 50 μM)	[137]
277	Toonasinemine E	*T. sinensis*	Root barks	HepG-2, MCF-7, and U2OS (IC_50_ > 50 μM) inhibitory in RAW 264.7 cell (inactive)	[137]
278	Toonasinemine F	*T. sinensis*	Root barks	HepG-2, MCF-7 and U2OS (IC_50_ > 50 μM) inhibitory in RAW 264.7 cell (IC_50_: 12.56 ± 0.62 μM)	[137]
279	Toonasinemine G	*T. sinensis*	Root barks	HepG-2, MCF-7, and U2OS (IC_50_ > 50 μM) inhibitory in RAW 264.7 cell (inactive)	[137]
280	Toonasin A	*T. sinensis*	Bark	Not reported	[138]
281	Toonasin B	*T. sinensis*	Bark	Not reported	[138]
282	Toonasin C	*T. sinensis*	Bark	HL-60 (IC_50_: 18.61 ± 0.14 μM), SMMC-7721 IC_50_: 19.55 ± 0.19 μM), A549 (IC_50_: 15.07 ± 0.13 μM), MCF-7 (IC_50_: 17.79 ± 0.15 μM),and SW480 (IC_50_: 12.47 ± 0.11 μM)	[138]
283	Toonaolide I	*Toona ciliata*	Twigs	NLRP3 (IC_50_: 4.2 and 3.9 ± 0.6 μM against LDH release and IL-1β secretion, respectively)	[139]
284	Toonaolide R	*T. ciliata*	Twigs	NLRP3 (IC_50_: 9.7 and 8.4 ± 0.7 μM against LDH release and IL-1β secretion, respectively)	[139]
285	Toonaolide X	*T. ciliata*	Twigs	NLRP3 (inactive)	[139]
286	Toononoid B	*T. ciliata*	Leaves and twigs	HL-60, MCF-7, SW-480, SMMC-7721, and A549 (inactive)	[141]
287	Toononoid D	*T. ciliata*	Leaves and twigs	HL-60, MCF-7, SW-480, SMMC-7721, and A549 (inactive)	[141]
288	Toononoid H	*T. ciliata*	Leaves and twigs	HL-60, MCF-7, SW-480, SMMC-7721, and A549 (inactive)	[141]
289	Toonanoronoid H	*T. ciliata*	Leaves and twigs	HL-60, MCF-7, SW-480, SMMC-7721, and A549 (inactive)	[141]
290	Toonaone I	*T. ciliata*	Twigs	NLRP3 (inactive)	[174]
291	Ciliatone C	*T. ciliata*	Twigs	Not reported	[140]
292	Ciliatone D	*T. ciliata*	Twigs	NLRP3 (IC_50_: 9.7 ± 3.7 μM and 7.8 ± 4.9 μM against LDH release and IL-1β secretion, respectively	[140]
293	Ciliatone E	*T. ciliata*	Twigs	Not reported	[140]
294	Ciliatone F	*T. ciliata*	Twigs	Not reported	[140]
295	Trichinenlide A	*Trichilia sinensis*	Leaves and twigs	Not reported	[142]
296	Trichinenlide F	*T. sinensis*	Leaves and twigs	Not reported	[142]
297	Trichinenlide G	*T. sinensis*	Leaves and twigs	Not reported	[142]
298	Trichiliasinenoid D	*T. sinensis*	Leaves and twigs	Inhibitory in RAW 264.7 cell (IC_50_: 93.8 μM) *S. aureus, P. aeruginosa, E.coli, and C. albicans* (MIC > 512 μg/mL)	[143]
299	Triconoid A	*Trichilia connaroides*	Leaves and twigs	Not reported	[144]
300	Triconoid B	*T. connaroides*	Leaves and twigs	Not reported	[144]
301	6α,7α-diacetoxy-3-oxo-24,25,26,27-tetranorapotirucalla-1,14,20(22)-trien-21,23-lactam	*Chisocheton paniculatus*	Fruits	LPS stimulated BV2 cells (69.0 ± 2.7% at 20.0 μM)	[145]
302	Walsunoid I	*Walsura robusta*	Leaves	Not reported	[146]
303	Aphanalide M	*Aphanamixis grandifolia*	Fruits	HL-60, Bel-7402, HepG2, SMMC-7721, A549, and MCF-7 (inactive)	[147]

### Nitrogen-containing triterpenoids

**Fig. 22:**
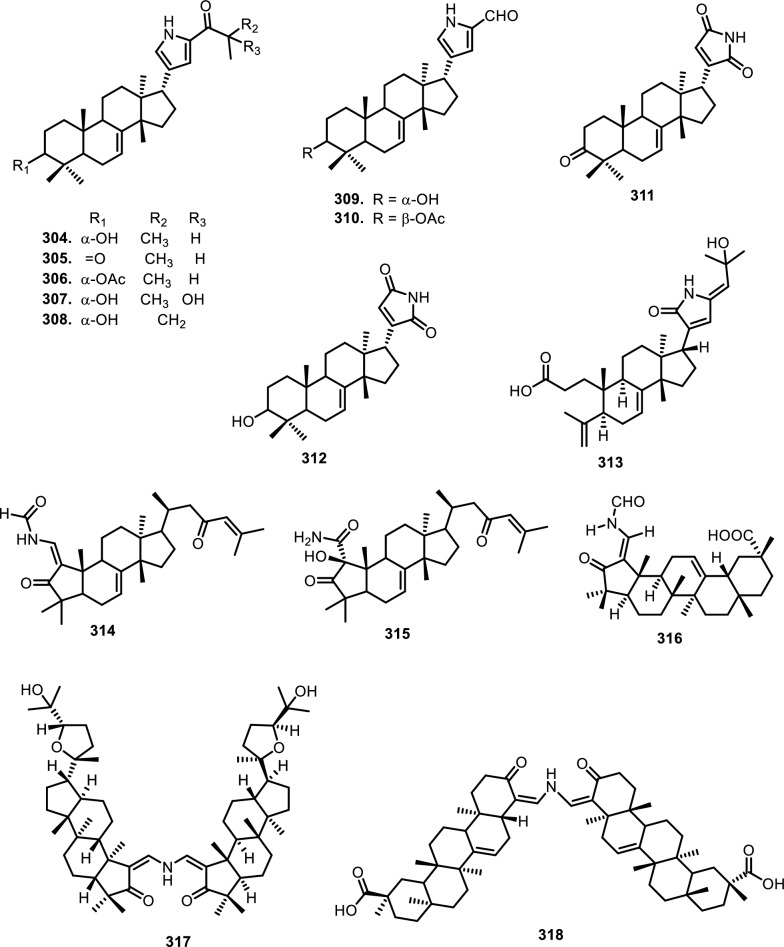
The chemical structures of compounds **304**–**318**

According to [148], eight tirucallane-type alkaloids, namely laxiracemosin A-H (**304–311**) were isolated from the bark of *Dysoxylum laxiracemosum* C.Y.Wu et H.Li. Additionally, compounds **304** and **311** were found in the stem bark of *Aphanamixis grandifolia* Blume [149]*.* Compound **311** was also reported in the leaves and twigs of *Dysoxylum lenticellatum* C.Y.Wu [150]. In compound **304**, the structure is defined by a hydroxyl group at the α-position (C-3), a methyl group at C-26, and a hydrogen atom at C-25. Compound **305** has a carbonyl group at the C-3 instead of the hydroxyl group, while **306** contains an acetoxy group at C-3. Compounds **307** and **308** retain the hydroxyl group at C-3, but with variations in the substitutions at C-25, **307** has a hydroxyl group, while **308** has a methylene group. Compound **309** still carries a hydroxyl group at the α-position (C-3), but **310** has a β-acetoxy group instead of the hydroxyl group. Finally, compound **311** features a carbonyl group at the C-3, with a carbonyl groups at C-21 and C-23. Another chemical analysis of the leaves and twigs *Dysoxylum lenticellatum* C.Y.Wu produced dysolenticin J (**312**), which has a similar structure to **311**. Compound **312** has a hydroxyl group at the C-3 instead of the carbonyl group [150]. [151] isolated congoensin A (**313**) from the bark of *Entandrophragma congoense* A.Chev*.* The phytochemical investigation of the stem of *Aphanamixis grandifolia* Blume resulted in the isolation of two tirucallane-type compounds, namely aphanamgrandin E (**314**) and F (**315**) [152]. An oleanane-type, which was dysoxyhainanin A (**316**), was isolated from the twigs and leaves of *Dysoxylum hainanense* Merr. [153]. Furthermore, two dimeric triterpenoids, namely silvaglenamin (**317**) were found in the root bark of *Aglaia silvestris* (M.Roem.) Merr. [154] and turraenine (**318**) was isolated from the leaves of *Turraea* sp (Table 4, Fig. 22).

**Table 4:** *N*-containing triterpenoids on Meliaceae family

No	Compounds	Species	Plant part	Biological activity	References
Tirucallane-type					
304	Laxiracemosin A	*Dysoxylum laxiracemosum*	Barks	HL-60 (IC_50_: 3.1 μM), SMMC-7721 (IC_50_: 9.5 μM), A549 (IC_50_: 5.4 μM), MCF-7 (IC_50_: 16.8 μM), and SW480 (IC_50_: 7.2 μM)	[148]
		*Aphanamixis grandifolia*	Stem barks	Not reported	[149]
305	Laxiracemosin B	*D. laxiracemosum*	Barks	HL-60 (IC_50_: 12.8 μM), SMMC-7721 (IC_50_: 19.0 μM), A549 (IC_50_: 13.4 μM MCF-7 and SW480 (inactive)	[148]
306	Laxiracemosin C	*D. laxiracemosum*	Barks	HL-60, SMMC-7721, A549, MCF-7, and SW480 (inactive)	[148]
307	Laxiracemosin D	*D. laxiracemosum*	Barks	HL-60 (IC_50_: 6.8 μM) SMMC-7721, A549, MCF-7, and SW480 (inactive)	[148]
308	Laxiracemosin E	*D. laxiracemosum*	Barks	HL-60 (IC_50_: 1.5 μM), SMMC-7721 (IC_50_: 2.7 μM), A549 (IC_50_: 3.7 μM), MCF-7 (IC_50_: 5.1 μM), and SW480 (IC_50_: 3.7 μM)	[148]
309	Laxiracemosin F	*D. laxiracemosum*	Barks	HL-60 (IC_50_: 15.7 μM) and SMMC-7721 (IC_50_: 15.6 μM) A549, MCF-7, and SW480 (inactive)	[148]
310	Laxiracemosin G	*D. laxiracemosum*	Barks	HL-60, SMMC-7721, A549, MCF-7, and SW480 (inactive)	[148]
311	Laxiracemosin H	*D. laxiracemosum*	Barks	HL-60, SMMC-7721, A549, MCF-7, and SW480 (inactive)	[148]
		*D. lenticellatum*	Leaves and twigs	HL-60, SMMC-7721 (inactive)	[150]
		*A. grandifolia*	Stem barks	Not reported	[149]
312	Dysolenticin J	*Dysoxylum lenticellatum*	Leaves and twigs	HL-60 and SMMC-7721 (inactive)	[150]
313	Congoensin A	*Entandrophragma congoënse*	Barks	*Plasmodium falciparum* NF54 (IC_50_: 5.5 μM); L6 (IC_50_: 10.6 μM)	[151]
314	Aphanamgrandin E	*A. grandifolia*	Stem	Not reported	[152]
315	Aphanamgrandin F	*A. grandifolia*	Stem	Not reported	[152]
Oleanane-type					
316	Dysoxyhainanin A	*Dysoxylum hainanense*	Twigs and leaves	*S. aureus* ATCC 25923, *S. epidermidis* ATCC 12228, *M. luteus* ATCC 9341, and *B. subtilis* CMCC 63501 (MIC 12.5, 6.25, 12.5, 6.25 μg/mL, respectively)	[153]
Dimeric triterpenoid					
317	Silvaglenamin	*Aglaia silvestris*	Root barks	Not reported	[154]
318	Turraenine	*Turraea* sp.	Leaves	FCM29 (16.6 μg/mL)	[167]

### Others

**Fig. 23:**
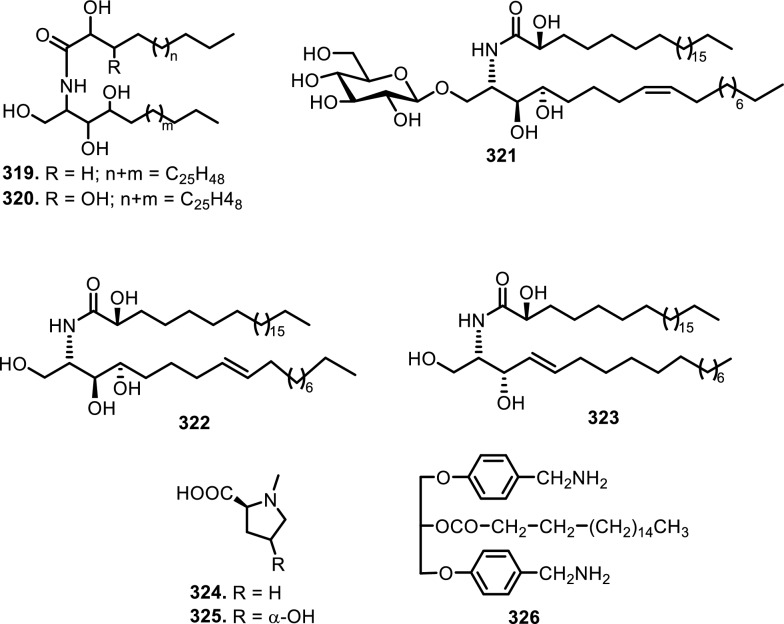
The chemical structures of compounds **319**–**326**

Research has identified five ceramides isolated from Meliaceae plant species. Ceramides A (**319**) and B (**320**) were isolated from the twigs of *Guarea mayombensis* Pellegr. [155]. Other ceramides, including 1-*O*-β-D-glucopyranosyl-(2*S*,3*S*,4*R*,8*Z*)-2-*N*-(2′-hydroxytetra cosanoyl)heptadecasphinga-8-ene (**321**), (2*S*,3*S*,4*R*,8*E*)-2-*N*-(2′-hydroxy tetra cosanoyl)-hepta decasphinga-8-ene (**322**), and (2*S*,3*R*,4*E*)-2-*N*-(2′-hydroxytetracosanoyl)-heptadecasphinga-4-ene (**323**) were isolated from the whole bodies of *Munronia henryi* Harms [132]. Moreover, nitrogen-containing compounds featuring a proline backbone were produced by the leaves of *Trichilia claussenii* C.DC. *N*-methylproline (**324**) has a nitrogen atom bonded to a methyl group, which is the defining feature of this compound, differentiating it from regular proline. In 4-hydroxy-*N*-methylproline (**325**), a hydroxyl group is attached at C-4 on the pyrrolidine ring in addition to the methylated nitrogen. This modification introduces polarity, which may affect the compound’s solubility and interactions in biological systems. The presence of the carboxyl group at the 2-position remains unchanged in both compounds, preserving their acidic properties. In addition, 1,3-di-benzene carbon amine-2-octadecylic acid-glyceride (**326**) was isolated from the twigs of *Carapa guianensis* Aubl. [156, 157] (Table 5, Fig. 23).

**Table 5:** *N*-containing others on Meliaceae family

No	Compounds	Species	Plant part	Biological activity	References
319	Ceramide A	*Guarea mayombensis*	Twigs	Not reported	[155]
320	Ceramide B	*G. mayombensis*	Twigs	Not reported	[155]
321	1-*O*-β-D-glucopyranosyl-(2*S*,3*S*,4*R*,8Z)-2-*N*-(2′-hydroxytetracosanoyl)heptadecasphinga-8-ene	*Munronia henryi*	Whole bodies	*P. brassicae* L (AR: 62.0%)	[132]
322	(2*S*,3*S*,4*R*,8E)-2-*N*-(2′-hydroxytetracosanoyl)-heptadecasphinga-8-ene	*M. henryi*	Whole bodies	*P. brassicae* L (AR: 3.0%)	[132]
323	(2*S*,3*R*,4E)-2-*N*-(2′-hydroxytetracosanoyl)- heptadecasphinga-4-ene	*M. henryi*	Whole bodies	*P. brassicae* L (inactive)	[132]
324	*N*-methylproline	*Trichilia claussenii*	Leaves	Not reported	[156]
325	4-hydroxy-*N*-methylproline	*T. claussenii*	Leaves	Not reported	[156]
326	1,3-di-benzene carbon amine-2-octadecylic acid-glyceride	*Carapa guaianensis*	Twig	Not reported	[157]

## Biological activities

The presence of nitrogen in bioactive natural products significantly influences their pharmacological properties. Nitrogen-containing compounds (N-compounds) often exhibit enhanced bioactivity due to their ability to participate in hydrogen bonding, ionic interactions, and molecular recognition processes with biological targets [158]. Nitrogen-containing functional groups, such as amines, amides, pyridines, and indoles, enhance molecular recognition by interacting with proteins, enzymes, and nucleic acids through hydrogen bonding and electrostatic interactions. This property is particularly evident in alkaloids, which frequently act on neurotransmitter receptors, ion channels, and enzymes. For example, β-carboline alkaloids interact with GABA receptors, contributing to their anxiolytic and sedative effects [159]. Similarly, nitrogen-containing flavaglines, such as rocaglamide, inhibit eukaryotic initiation factor 4A (eIF4A), thereby suppressing cancer cell proliferation. The ability of nitrogen-containing flavaglines to interfere with cellular translation makes them valuable anticancer leads.

Although Meliaceae family produces numerous nitrogen-containing compounds, there is no comprehensive discussion of their biological activities in the phytochemical literature. These compounds have a wide range of bioactivities, including cytotoxic effects against different cancer cell lines, insecticidal, anti-inflammatory, molluscicide, antibacterial, antimalarial, tyrosinase inhibition, osteoclast differentiation inhibition, and α-glucosidase inhibition activity. The diverse bioactivities underscore their importance in medicinal research and potential for developing new therapeutic agents.

### Cytotoxic effects

Numerous alkaloids that have been isolated from Meliaceae family have been evaluated for cytotoxicity with human cancer cell lines. These compounds included amocurine A (**20**), B (**21**), and C (**22**), which showed significant antiproliferative activity against various cancer cells using the MTT assay. Cells were incubated with compounds for 72 h, and IC_50_ values were determined from three independent experiments. Compounds were dissolved in DMSO, with a final DMSO concentration not exceeding 0.5%. In alkaloids **20, 21, 25,** and **26** were inactive against KB, and MCF-7, while **22** had significant activity against KB (IC_50_ = 3.5 ± 0.6 µM), and MCF-7 (IC_50_ = 4.2 ± 1.4 µM), as compared to the positive control doxorubicin with IC_50_ value of 0.5 ± 0.1 and 6.8 ± 0.1 μM, respectively. These alkaloids showed potential as antiproliferative agents targeting different cancer cell lines and suggest that the aporphine alkaloid’s aromatic ring system (C_6a_ = C_7_ bond), 1,2-methylenedioxy ring, and the structure’s planarity significantly influence antiproliferative activity. Amocurine C (IC_50_ = 6.7 µM) exhibited notable cytotoxicity against NCI-H187 cells, while amocurine B showed slightly lower potency (IC_50_ = 40.2 µM). This activity was appreciable, though not as strong as the positive controls (doxorubicin and ellipticine) with IC_50_ values of 0.7 ± 0.2 µM and 1.7 ± 0.1 µM, respectively. The findings highlight the potential of structural elements, such as the aporphine alkaloid's aromatic ring system and methylenedioxy ring, in enhancing cytotoxic properties [28, 45].

Compound **24** showed antiproliferative effects against KB cells (IC_50_ = 9.3 ± 0.8 µM), MCF-7 (IC_50_ = 10.1 ± 0.2 µM), and NCI-H187 (IC_50_ = 8.5 ± 0.5 µM) cancer cells, while **25** and **26** showed more modest activity against NCI-H187 cancer cells (IC_50_ = 30.4 ± 1.1 and 25.2 ± 0.8 µM). Alkaloids isolated from *Amoora cucullata* Roxb. could have potential as cancer drug leads [28, 45].

Transitioning to chromone alkaloids, rohitukine (**32**), isolated from *Dysoxylum binectariferum*, has garnered attention as the natural precursor of flavopiridol, a synthetic CDK inhibitor currently in Phase III clinical trials. Rohitukine demonstrated moderate cytotoxicity against HL-60 (leukemia), HCT-116 (colon cancer), SKOV3 (ovarian cancer), and MCF-7 cells, with IC₅₀ values ranging from 7.5 to 20 µM. These values were obtained using both sulforhodamine B (SRB) and MTT assays after 48 or 72 h of incubation. Although its potency is lower than flavopiridol, rohitukine remains a key lead compound for developing CDK-targeted therapies [55, 56]. Further expanding the diversity of active compounds, dysoline (**45**), also from *D. binectariferum*, displayed selective and potent cytotoxicity against HT1080 fibrosarcoma cells, with an IC₅₀ value of 0.21 µM as determined via the MTT assay. Interestingly this compound appeared to show selectivity being apparently inactive on Colo205, HCT116, NCIH322, A549, MOLT-4, and HL60 cells at concentrations greater than 10 μM. Dysoline did not show activity against various kinases such as PKB-β, CDK2/A, CDK9/T1, Aurora A, Aurora B, AMPK (hum), CK12, CK1, DYRK1A, IGF-1R, IR, and VEGFR1 therefore its mechanism of action remains to be elucidated [33].

Among the most studied cytotoxic agents in this family are flavaglines, a unique group of cyclopenta[b]benzofurans primarily isolated from *Aglaia* species. For instance, rocaglamide (**115**) exhibited strong antiproliferative effects across KB, A549 (lung carcinoma), and HL-60 cells, with IC₅₀ values typically in the nanomolar range (e.g., 0.01–0.08 µM), as determined via MTT assays conducted over 48–72 h. Aglapervirisin I (**157**) has been shown to have significant cytotoxic activity against various cancer cell lines, making it a promising candidate for further research in cancer treatment. IC_50_ values were reported to be 27.7 μM against HepG2 liver cancer cells, 29.4 μM against HL-60 leukaemia cells, 36.0 μM against MCF-7 breast cancer cells, and 23.1 μM against HT-29 colon cancer cells. Meanwhile, compounds **157** and **115** [26, 93, 160].

Based on the results, 3'-hydroxy-desmethylrocaglamide (**128**) showed higher activity against the K562 cell line with IC_50_ value of 4.5 μg/mL, compared to desmethylrocaglamide (**121**), which had IC_50_ value of 9.5 μg/mL. Compounds **126** and **127** were inactive on K562 cancer cell [107]. Didesmethylrocaglamide (**124**) showed significant activity against HT-29 (ED_50_ = 0.021 μM) [27]. Compound **132** showed cytotoxic effects against HeLa (IC_50_ = 0.32 μM), SGC7901 (IC_50_ = 0.12 μM), and A549 (IC_50_ = 0.25 μM) cancer cells [35]. These findings suggest that the presence of the OH group at C-8b is essential for enhancing the cytotoxic activity of the compound [27, 107].

Furthermore, aglaroxin A (**135**) is derivative featuring a cyclopentatetrahydrobenzofuran core, which is characteristic of rocaglamides from the *Aglaia* genus. It has a methylenedioxy group at C-6 and C-7 instead of the more common C-6 methoxy unit, influencing its biological activity and derivative, aglaroxin A 1-*O*-acetate (**136**), showed significant cytotoxic activity against various cancer cell lines. Compound **135** showed cytotoxic effects with ED_50_ values of 0.04 μg/mL against Lu1 lung cancer cells, 0.02 μg/mL against LNCaP prostate cancer cells, 0.06 μg/mL against MCF-7 breast cancer cells, and 0.1 μg/mL against HUVEC endothelial cells. In comparison, **136** showed greater potency with ED_50_ values of 0.001 μg/mL against Lu1, 0.01 μg/mL against LNCaP, 0.02 μg/mL against MCF-7, and 0.5 μg/mL against HUVEC cell line. These results showed the increased efficacy of the acetate derivative in inhibiting cancer cell growth [62]. Meanwhile, dehydroaglaiastatin (**141**) showed significant cytotoxic activity across a wide range of cancer cell lines, such as A549, HL-60, HT-29, KB, and P-388 (ED_50_ = 0.0012, 0.0010, 0.0015, 0.01, 0.0018 µM, successively) cancer cell lines, as compared to the positive control mithramycin with ED_50_ values of 0.08, 0.07, 0.09, 0.07, and 0.06 μg/mL, respectively. The presence of a cyclopentapyrimidinone subunit in the structure of compound **141** makes this compound highly cytotoxic against HepG2 cancer cells (IC_50_ = 0.69 ± 0.06 μM). Compound **141** also showed cytotoxic activity against HEL (IC_50_ = 0.03 ± 0.001 μM) and MDA-231 (IC_50_ = 1.06 ± 0.27 μM) cancer cells, as compared to the positive control adriamycin with IC_50_ values of 0.17 ± 0.02 and 0.54 ± 0.08 μM, respectively. However, 3'-hydroxyaglaroxin C (**143**) showed lower cytotoxic activity compared to **141**, specifically against HEL cancer cells, with an IC_50_ value of 0.17 ± 0.06 μM. These results indicate that the addition of an OH group on the phenyl ring could decrease its cytotoxic activity against HEL cells [34, 161, 162].

Compounds **142** and **168** were evaluated using in vitro assays against A549 (lung), HL-60 (leukemia), HT-29 (colon), KB (nasopharyngeal), and P-388 (murine leukemia) cell lines. Compound **142** exhibited strong cytotoxicity with ED₅₀ values of 0.014 μg/mL (A549), 0.012 μg/mL (HL-60), 0.011 μg/mL (HT-29), 0.025 μg/mL (KB), and 0.012 μg/mL (P-388). In comparison, the reference drug mithramycin displayed ED₅₀ values of 0.08, 0.07, 0.09, 0.07, and 0.06 μg/mL, respectively, across the same cell lines. However, compound **168** showed markedly lower activity and was inactive against HL-60, HT-29, and KB cells under similar test conditions [34, 72, 161].

In a separate study, compound **151** demonstrated potent cytotoxicity against HT-29 (colon) and PC-3 (prostate) cancer cell lines, with IC₅₀ values of 2.3 μM for both, as measured by the MTT assay after 72 h of exposure [111]. Additionally, several compounds including **142, 90, 91, 168, 92**, and **93** were assessed for activity against the P-388 cell line. Among these, compound **142** again showed superior potency (ED₅₀ = 0.012 μg/mL), while compounds 90, 91, and 168 yielded ED₅₀ values of 3.62, 3.86, and 3.41 μg/mL, respectively. Compounds **92** and **93** exhibited moderate cytotoxicity with IC₅₀ values of 7.6 and 8.5 μg/mL [65, 72, 88]. The result of compound **74** test against KB-V1 had ED_50_ 10 μg/mL. Gigantamide A (**83**) was highly promising to HT-29 (ED_50_ = 0.021 μM), when **167** was inactive to P-388 and MOLT-4 but active to HEL (IC_50_ = 6.25 ± 0.26 μM) and MDA-231 (IC_50_ = 12.51 ± 0.31 μM) cancer cells, as compared to the positive control adriamycin with IC_50_ values of 0.17 ± 0.02 and 0.54 ± 0.08 μM, respectively [27, 34, 65, 77].

The difference in cytotoxic activity between nitrogen-containing and non-nitrogen-containing compounds in the study can be attributed to structural variations that influence their biological interactions. Rocagloic acid, which does not contain nitrogen, exhibited significantly higher cytotoxicity across multiple cancer cell lines, with ED_50_ values in the nanogram range. In contrast, the nitrogen-containing compounds, **168** and **90**, showed much weaker activity, often exceeding 50 µg/mL in most cell lines. This difference is likely due to the presence of nitrogen-based functional groups, such as amides and heterocyclic rings, which can alter the compound’s electronic distribution, molecular conformation, and ability to interact with biological targets. Additionally, the nitrogen groups may increase hydrophilicity, affecting the compound’s ability to permeate cell membranes and reach intracellular targets effectively. Furthermore, rocagloic acid’s rigid and planar structure may enhance its interaction with biological macromolecules, whereas the flexibility introduced by nitrogen-containing moieties in elliptifoline and elliptinol could reduce their binding affinity. These factors together contribute to the observed differences in cytotoxic potency, as supported by the study conducted by [72].

Aglaxiflorin C (**182**) has no activity on P-388 and MOLT-4 cell lines, indicating its limited anticancer potential under the tested conditions [77]. Similiarly, aglaodoratin A (**189**), B (**190**), and F (**194**), isolated from the leaves of *Aglaia odorata* Lour., were found to be inactive against MG-63, HT-29, and SMMC-7721 cancer cells, with IC_50_ values greater than 10 μM. However, aglaodoratins C-E (**191–193**) showed selective cytotoxicity against cancer cells. Notably, compound **191** exhibits a significant cytotoxic activity against MG-63 (IC_50_ = 1.2 μM) and HT-29 (IC_50_ = 0.097 μM) cancer cells, as compared to the positive control taxol with IC_50_ values of 0.832 and 0.002 μM, respectively but was inactive against SMMC-7721 (IC_50_ > 10 μM). Aglaodoratin D (**192**) showed cytotoxic activity specifically against MG-63 cancer cells (IC_50_ = 0.75 μM) but was inactive against HT-29 and SMMC-7721 cells (IC_50_ > 10 μM). Aglaodoratin E (**193**) showed cytotoxic activity against SMMC-7721 cancer cells (IC_50_ = 6.25 μM), as compared to the positive control taxol with IC_50_ value of 3.1 μM but was inactive against MG-63 and HT-29 (IC_50_ > 10 μM) [113]. Compound **90** was tested on the HL-60 cell lines with an ED_50_ of 32.1 μg/mL but was inactive against A549, HT-29, and KB cell lines. Compounds **74, 77**, and **78** were very promising against KB-V1* cells (resistant in 1 μg/mL vinblastine) with ED_50_ values of 8.5, 6.4, and 4.2 μg/mL, respectively [65, 72].

Further investigations into compounds **196–198** showed varied cytotoxic activity against different cancer cell lines. Compound **196** showed cytotoxic activity against HEL cancer cells (IC_50_ = 8.40 ± 0.85 μM), as compared to the positive control adriamycin with IC_50_ value of 0.17 ± 0.02 μM but was inactive against MDA-231 cells (IC_50_ > 20 μM). Compounds **197** and **198** were inactive against both HEL and MDA-231 cells (IC_50_ > 20 μM) suggesting that the presence of two OH groups on the benzene ring may reduce cytotoxic activity. Compound **222** was inactive against HeLa, SGC7901, and A549 cancer cells [34, 35]. Compared to the research by [62], compounds **199–203** and **231** were inactive on Lu1, LNCaP, MCF-7, and HUVEC cancer cells (ED_50_ > 5 μg/ mL).

Compounds **206–209** and **212** showed varying degrees of cytotoxic activity against Lu1, LNCaP, and MCF-7 cancer cells. Specifically, compound **206** showed cytotoxic activity with ED_50_ values of 1.8 μM for Lu1, 1.4 μM for LNCaP, and 1.8 μM for MCF-7 cancer cells, as compared to the positive control paclitaxel with ED_50_ values of 0.0023, 0.0047, and 0.0007 μM, respectively. Compound **212** was moderate with ED_50_ values of 17.5 μM for Lu1, 21 μM for LNCaP, and 16.1 μM for MCF-7. Compound **208** also showed moderate activity with ED_50_ values of 18.1 μM for Lu1, 16.0 μM for LNCaP, and 13.5 μM for MCF-7. Meanwhile, compounds **207** and **209** were inactive against all three cell lines (ED_50_ > 20 μM). This study has demonstrated that certain derivatives of cyclopenta[b]benzopyran may exhibit cytotoxic properties, influenced by the positions of substituents at C-3 and C-4, type of the amide moiety, and the orientation of the OH-10 [66].

Similar to **209**, compounds **213**, **214**, **215**, and **218** have no activity against HepG2, HL-60, MCF-7, and HT-29 cancer cells, with IC_50_ values greater than 50 μM. However, compound **216** showed significant cytotoxic activity against HepG2 (IC_50_ = 10.9 μM), HL-60 (IC_50_ = 2.2 μM), MCF-7 (IC_50_ = 8.5 μM), as compared to the positive control *cis*-platinum with IC_50_ values of 8.2, 2.5, and 6.4 μM, respectively. This compound was also found to show cytotoxicity against HT-29 cells (IC_50_ = 1.4 μM), as compared to the positive control taxol with IC_50_ value of 0.002 μM [114]. Compound **219** had no potential against HT-29 cancer cells (ED_50_ > 10 μM), while its C-10 epimer **220** showed significant activity (ED_50_ = 0.46 μM) [27]. Compound **239** denoted inactive against A549, RERF, PC-3, PC-6. QG-56, and QG-90 cell lines (IC_50_ > 100 µM) (Wu et al., 2014). **248** was non-active on HepG2 and MCF-7 cell lines with IC_50_ > 50 μM [128].

Compounds **242** and **243** showed cytotoxic activity against MDA-MB-231 cancer cells with IC_50_ values of 2.70 ± 0.63 μM and 15.73 ± 6.07 μM, respectively, as compared to the positive control cisplatin with IC_50_ value of 1.70 ± 0.43 μM [124, 125]. Meanwhile, compounds **249–254** have been tested for MDR reversal ability on MCF-7/DOX cells, showing inactive results and were unable to overcome multidrug resistance in breast cancer cells [129, 130].

Based on the analysis, compounds **269** and **270** were inactive against Hela and A549 cancer cells. Compound **273** showed cytotoxic activity against HepG-2 cancer cells with an IC_50_ of 40.67 ± 3.23 μM and was inactive on MCF-7 as well as U2OS cancer cells (IC_50_ > 50 μM). Compound **274**, **275**, and **277–279** were inactive on HepG-2, MCF-7, and U2OS cancer cells (IC_50_ > 50 μM). Meanwhile, compound **276** showed activity against HepG-2 (IC_50_ = 11.63 ± 1.02 μM) and MCF-7 (IC_50_ = 36.77 ± 2.79 μM) cancer cells, as compared to the positive control doxorubicin with IC_50_ values of 3.25 ± 0.29 and 1.93 ± 0.15 μM, respectively but was inactive on U2OS. Compound **282** showed significant cytotoxic activity against a range of cancer cell lines. The IC_50_ values for HL-60, SMMC-7721, A549, MCF-7, and SW480 were 18.61 ± 0.14, 19.55 ± 0.19, 15.07 ± 0.13, 17.79 ± 0.15, and 12.47 ± 0.11 μM, respectively. This showed that compound **282** has considerable potential as an anticancer agent, showing the highest activity against the SW480 cell line. Other compounds, including **286**, **287**, **288**, and **289**, isolated from *Toona ciliata* M.Roem. leaves and twigs, did not show cytotoxic activity against cancer cell lines HL-60, MCF-7, SW-480, SMMC-7721, and A549, respectively. This showed that the compounds lacked potential as anticancer agents [141].

Cytotoxic activity was shown by compounds **304–309** against several cancer cell types. Specifically, compounds **304** and **308** showed efficacy against A549, MCF-7, HL-60, SMMC-7721, and SW480. Compound **305** showed activity against A549, HL-60, and SMMC-7721. In comparison to HL-60, compound **307** was effective, while **309** showed efficacy in combating HL-60 and SMMC-7721. Against every cell line tested, compounds **310**, **311**, **312**, and **108** showed no cytotoxic effect [148, 150, 151]. The Meliaceae family provides a diverse range of bioactive alkaloids and flavaglines with significant cytotoxic effects across various cancer cell lines. The SAR insights gained highlight specific structural features essential for maximizing anticancer activity, making these compounds prime candidates for further development as therapeutic agents.

### Insecticidal activity

Numerous compounds from the Meliaceae family, particularly rocaglamide and its analogues, have been investigated for insecticidal properties against agriculturally significant pests. The larvicidal and growth-inhibitory activities were evaluated primarily through contact or ingestion bioassays, with effective concentration (EC₅₀), lethal concentration (LC₅₀), and lethal dose (LD₅₀) reported as outcome measures. Initial screening of piriferinol (**75**), edulimide (**76**), and cyclorocaglamide (**114**) against *Spodoptera littoralis* showed no activity, with LC₅₀ and EC₅₀ values exceeding 250 µg/g fresh weight [74, 163]**.** The efficacy of rocaglamide (**115**) and aglaroxins A (**135**), B (**138**), F (**139**), D (**140**), E (**118**), C (**146**), G (**147**), H (**148**), I (**149**), K (**163**), L (**167**), J (**169**) was evaluated against four insect species, namely *Heliothis virescens*, *Spodoptera littoralis*, *Plutella xylostella*, and *Diabrotica balteata*. The rocaglamide compound **115** showed high efficacy with an effective dose of 3 mg/L against all species. However, there was the exception of *Heliothis virescens* at the L3 larval stage, where the effective dose was 12.5 mg/L. This compound also showed significant insecticidal activity in *Peridroma saucia* (EC_50_ = 0.91 ppm; LD_50_ = 0.28 ppm) [36].

Among the aglaroxins, aglaroxin A (**135**) stood out, with effective doses of 0.8 mg/L against early and late instar *H. virescens*, and 3 mg/L against *S. littoralis, P. xylostella*, and *D. balteata*. For *H. virescens* and *S. littoralis* at the L3 stage, 12.5 mg/L was required. Aglaroxin B (**138**) displayed moderate efficacy: 3 mg/L was effective against *H. virescens* (e/l and L1 stages), and 12.5 mg/L was needed against *S. littoralis* (L3 stage). However, minimal effect was seen against *P. xylostella* (12.5 mg/L) and no activity against *D. balteata* even at > 100 mg/L [37].

Aglaroxin E (**117**) showed comparable activity to aglaroxin B (**138**) against *Heliothis virescens* and *Spodoptera littoralis* but had enhanced efficacy against *Plutella xylostella.* There was no significant effect against *Diabrotica balteata* at doses exceeding 100 mg/L. Notably, compounds **117** and **135** showed insecticidal activity against *Spodoptera littoralis* (LC_50_ = 1.0 ± 0.35 ppm; EC_50_ = 0.09 ± 0.03 ppm). In contrast, aglaroxin F (**139**) required higher doses (25–50 mg/L) for efficacy against *H. virescens, S. littoralis*, and *P. xylostella*, but was more potent against *D. balteata* (12.5 mg/L). Aglaroxin C (**146**) was moderately active (3 mg/L) against *H. virescens* at early stages, with reduced efficacy at later stages and poor activity (> 100 mg/L) against *S. littoralis* (L3). Activity against *P. xylostella* was moderate (12.5 mg/L), with no significant effect on *D. balteata*. Further screening of aglaroxins D (**140**), G (**147**), H (**148**), I (**149**), J (**169**), K (**163**), and L (**167**) showed minimal activity or were ineffective at doses exceeding 100 mg/L. These compounds were found to be effective against almost all species and larval stages tested. Compound **147** showed efficacy against *Diabrotica balteata* at a dose of 50 mg/L and **149** was active at 12.5 mg/L [37].

Rocaglamide D (**116**) had less insecticidal activity against *Spodoptera littoralis* with LC_50_ 1.5 ± 0.65, EC_50_ 0.21 ± 0.08 ppm than **117** (LC_50_ = 1.0 ± 0.35; EC_50_ = 0.09 ± 0.03 ppm), as compared to the positive control azadirachtin with LC_50_ and EC_50_ value of 0.9 ± 035 and 0.04 ± 038 ppm, respectively [38], while **120** and **123** were nonactive [105]. Compound **121** was active against *Peridroma saucia* (LD_50_ = 1.06 ppm; EC_50_ = 2.01 ppm) [36] and *Spodoptera littoralis* (LC_50_ = 1.3 ppm; EC_50_ = 0.27 ppm) [104]. When *Spodoptera littoralis* was evaluated with compounds **118**, **119**, and **122**, the results showed LC_50_ values of 7.1, 8.0, and 8.1 ppm, respectively [104, 110]. The decrease in insecticidal activity can be attributed to the presence of an OCOCH_3_ group at C-1, with similar results also demonstrated in studies of other rocaglamide derivatives isolated from *Aglaia elliptica* [103, 110]. Further structure–activity insights emerged with compounds **129**, **131**, and **141** were evaluated against *Spodoptera littoralis,* showing good activity with LC_50_ = 1.6 ± 0.55, 1.97, and 1.6 ppm, EC_50_ = 0.21 ± 0.07, 0.14, and 0.4 ppm [38, 105]. Compared to the three previous compounds, **153** against *Spodoptera littoralis* showed a smaller LC_50_ value of 6.52 ppm [164].

Previous studies have shown that the benzofuran skeleton is key to the insecticidal activity of rocaglamide derivatives, as structurally similar analogues, known as aglains, lack insecticidal activity due to the presence of a pyran ring instead of a furan ring. Substituents attached to the C-8b position of the furan ring influence the insecticidal activity of rocaglamide derivatives. Rocaglamide derivatives are potent natural insecticides and could serve as promising lead compounds for plant protection. The insecticidal activities of these Meliaceae-derived compounds present promising avenues for the development of natural pesticides. Their potent, low-dose efficacy against multiple economically relevant pests makes them ideal candidates for sustainable pest management in agriculture. Given the ongoing need to reduce synthetic pesticide use and manage resistance, these natural compounds offer an eco-friendly alternative that could help safeguard crop health while mitigating environmental and health impacts.

Experimental evidence supports this observation, as cytotoxicity studies on *Aglaia elliptifolia* demonstrated that rocagloic acid (non-nitrogen-containing) exhibited significantly stronger cytotoxic effects compared to nitrogen-containing compounds like elliptifoline and elliptinol. Similarly, insecticidal studies on *Aglaia oligophylla* revealed that non-nitrogenous rocaglamide derivatives, which maintain the furan ring structure, were more effective against *Spodoptera littoralis* larvae, whereas nitrogen-containing aglain-type compounds lacked insecticidal activity. The substitution of functional groups also plays a crucial role in bioactivity, as studies found that replacing hydroxyl groups with methoxy or ethoxy groups at certain positions led to a complete loss of insecticidal potency. Overall, the presence of nitrogen affects the bioavailability, membrane permeability, and interaction of these compounds with biological targets. Non-nitrogen-containing rocaglamides exhibit higher cytotoxic and insecticidal activity due to their optimized structural configuration, which facilitates stronger interactions with biomolecules. These findings highlight the importance of molecular structure in determining the biological efficacy of natural compounds derived from Aglaia species [165].

### Anti-inflammatory

Members of the Meliaceae family have yielded a wide array of compounds with promising anti-inflammatory activity, primarily evaluated through their ability to inhibit key pro-inflammatory mediators such as TNF-α, IL-6, NO, and NF-κB. These effects were assessed using various in vitro assays on THP-1, RAW 264.7, and BV2 macrophage cell lines, employing methods such as enzyme-linked immunosorbent assay (ELISA) for cytokine quantification, the Griess reaction for nitric oxide (NO) measurement, and luciferase reporter assays for NF-κB inhibition. Methanol extracts of *Dysoxylum binectariferum* Hook.f. ex Bedd. fruits and leaves showed significant inhibition of TNF-α and IL-6 in THP-1 cells, with inhibition ranging from 43–60% at 2.5–3.12 μg/mL, as measured by ELISA suggesting the presence of potent anti-inflammatory constituents. Among isolated compounds, rohitukine (**32**) showed the most notable activity, suppressing TNF-α and IL-6 levels by 50% and 82%, respectively, at 5 μM. Similarly, chrotacumine K (**44**) displayed significant inhibition at 10 μM, while chrotacumine E (**38**) showed moderate effects [39]. From the stem bark of the same species, compound (45) exhibited strong activity, reducing TNF-α and IL-6 levels by 47% and 83%, respectively, at 0.1 M [33] In the genus Aglaia, anti-inflammatory activities were assessed on RAW 264.7 macrophages using the Griess assay to quantify NO production. Several compounds demonstrated moderate activity, including **61** (IC₅₀ = 65.3 μg/mL), **62** (83.4 μg/mL), **88** (19.5 μg/mL), **89** (12.3 μg/mL), **87** (7.4 μg/mL), **78** (8.6 μg/mL), and **91** (50.2 μg/mL) [27, 63]. In contrast, compounds **63**, **83**, and **124** were inactive. From *Melia azedarach* L., an indole alkaloid, methyl indole-3-carboxylate (**110**), showed moderate NO inhibition in RAW 264.7 cells, with an IC₅₀ of 42.2 ± 2.7 μM, as determined by the Griess assay [95].

More potent activity was observed in specific *Aglaia* compounds targeting the NF-κB pathway. For instance, compound **154** showed strong inhibition of NF-κB activation with an IC₅₀ of 0.06 μM [112]. Similarly, aglapervirisin J-M (**158–161**) displayed moderate inhibition in RAW 264.7 cells, with IC₅₀ values ranging from 29.5 to 43.7 μM [26]. Aglaxiflorin D (**167**) (IC_50_ = 2.1 µM) showed good anti-inflammation against RAW 264.7 cells [40], while **220** had significant activity on NF-kB with ED_50_ 2.4 µM [27]. Further research on anti-inflammatory compounds derived from Meliaceae family members has yielded promising results. Compounds **240**, **248**, and **263** have shown moderate efficacy, while **253–256**, **258**, and **262** were inactive against RAW 264.7 cells [122, 128, 130, 131].

In another investigation, several toonasinemine compounds were subjected to further analysis to ascertain the inhibitory impact on NO production in RAW 264.7 macrophages. Toonasinemine A (**273**), B (**274**), and F (**278**) showed significant efficacy against RAW 264.7 cells, with an IC_50_ value of 10.21–20.05 µM, as compared compared to the positive control L-NMMA with IC_50_ value of 32.55 ± 2.15 μM, while Toonasinemine E (**277**), and G (**279**) were inactive. Trichiliasinenoid D (**298)** showed weak NO inhibition in RAW 264.7 macrophages. Additionally, *Toona sinensis* (A.Juss.) M.Roem. and *Toona ciliata* M.Roem. also yielded three compounds, namely toonaolides (**283–285**), Toonaone I (**290**), and ciliatone D (**292**) which had been tested for anti-inflammatory. Compounds **283**, **284**, and **292** showed significant efficacy against NLRP3, with IC_50_ values of 4.2, 9.7, and 9.7 µM, respectively, while **285** and **290** had no discernible activity [139]. Anti-inflammatory testing was also conducted on BV2 cells and compound **301**, which showed effective inhibition at a concentration of 20 µM [145]. The diversity in anti-inflammatory efficacy across these compounds highlights several potent candidates, such as Rohitukine (**32**), Aglaxiflorin D (**167**), and compound **154**, which exhibited significant inhibition of inflammation at low concentrations. Compounds that target key pathways like NF-κB and NO production in macrophages show promise for further development in anti-inflammatory drug research. This data suggests that the Meliaceae family offers multiple bioactive compounds with potential for treating inflammatory diseases, paving the way for natural and targeted anti-inflammatory therapies.

### Molluscicide activity

The methanol extract of Dysoxylum lenticellare Gillespie leaves was evaluated for molluscicidal activity using a standard WHO-recommended bioassay, which measures snail mortality following exposure to plant extracts or compounds. The extract showed 100% mortality against Biomphalaria glabrata at a concentration of 100 ppm, indicating strong molluscicidal potential. Further testing of isolated alkaloids revealed that 3-epischelhammericine (**2**), 2,7-dihydro homoerysotrine (**3**), and 3-*epi*-12-hydroxy schelhammericine (**4**) showed the strongest molluscicidal activity, achieving 100% mortality at concentrations of 10 ppm or lower. Other compounds such as dyshomerythrine (**1**), 3-epi-2,18-dimethoxy-schelhammericine (**5**), homolaudanosine (**10**), and dyssoxyline (**11**) showed moderate but significant molluscicidal effects, while lenticellarine (**7**) and dysazecine (**12**) exhibited minimal activity even at the highest test concentrations. All compounds were assessed via snail immersion assays using Biomphalaria glabrata as the test organism. The strong activity of compounds **2–4** underscores their potential for development as eco-friendly molluscicides, which are crucial for schistosomiasis vector control [41–43].

### Antibacterial, antimalarial, and antifungal activity

Amocurine D (**23**) was successfully obtained from *Amoora cucullata* Roxb. and evaluated for antibacterial activity against *Staphylococcus aureus* and *Bacillus subtilis*, evaluated via the broth microdilution method. The results showed that the compound had good activity with a minimum inhibitory concentration (MIC) of 3.1 µg/mL [44]. Meanwhile, amocurine E (**27**) was evaluated only against *Bacillus subtilis* and showed the same MIC value as compound **23**. In comparison, compound **298** was subjected to testing against *Staphylococcus aureus, Pseudomonas aeruginosa, Echerichia coli*, and *Candida albicans.* The result obtained an MIC value of > 512 g/mL, confirming that **298** was not active against the bacteria. Nitrogen-containing alkaloids, such as indole and pyrrole derivatives, show strong antibacterial and antifungal activities by disrupting microbial cell membranes and inhibiting key metabolic enzymes. Non-N-compounds, such as phenolic compounds and terpenoids, exhibit antimicrobial effects primarily through oxidative stress induction and membrane disruption rather than enzymatic inhibition. For example, xylogranatine, a nitrogen-containing limonoid, has been shown to inhibit bacterial DNA gyrase, while limonoids without nitrogen lack this targeted inhibitory effect [166].

For antimalarial screening, compound **23** exhibited in vitro antiplasmodial activity against *Plasmodium falciparum* K1 strain with an IC₅₀ of 3.52 µM, assessed via the [^3^H]-hypoxanthine incorporation assay. Compound **28** demonstrated stronger activity (IC₅₀ = 1.84 µM). Additionally, compounds **313** and **318** were tested against Plasmodium falciparum strains NF54 and FCM29, yielding IC₅₀ values of 5.5 µM and 16.6 µg/mL, respectively, using the SYBR Green I fluorescence-based assay [45, 151, 167].

### Other activities

Compound **38** had been subjected to testing for tyrosinase inhibition activity using the mushroom tyrosinase enzyme assay, yielding 29.2% inhibition at 100 µg/mL, outperforming compound **39** which showed 25.8% inhibition at the same concentration [59]. Compounds **40** and **43** were evaluated for their effects on osteoclast differentiation, measured using TRAP (tartrate-resistant acid phosphatase) staining and enzyme assays, with IC₅₀ values of 9.8 µM and 15.1 µM, respectively. Meanwhile, nimbandiolactam-21 (**244**) was tested for α-glucosidase inhibition via a p-nitrophenyl-α-D-glucopyranoside (pNPG) colorimetric assay, showing moderate activity with IC₅₀ = 79.7 µM [60, 125].

## Conclusion

In conclusion, this research showed the significance of identifying new compounds from natural products to expand the sources of therapeutic molecules. Based on the results, Meliaceae family was identified as one of the plants with the largest source of therapeutic molecules, comprising more than 50 genera and 1400 species. Several investigations from various Asian and Indonesian nations had been carried out to explore these species, where nitrogen-containing compounds were isolated from Meliaceae. These molecules showed structural variety, which was described and proven valuable in the hunt for new compounds and diverse properties. In this research, several groups of nitrogen*-*containing compounds isolated from Meliaceae family were discussed. These included alkaloids and derivatives (113), flavaglines and derivatives (118), limonoids and derivatives (72), terpenoids and derivatives (15), as well as compounds from other groups (8). Previous research stated that nitrogen-containing compounds had cytotoxic, insecticidal, antibacterial, antimalarial, anti-inflammatory, molluscicide, and antiviral effects. According to the results, these nitrogen-containing compounds received widespread attention for their experimental outcomes on various cancer cells as well as anti-inflammatory properties. This research also discussed the compounds that could be used as medication candidates and suggested further activity testing into their mechanisms of action and therapeutic potential. Future research should prioritize synthetic modifications and clinical evaluations using in vivo models, followed by toxicological analysis to identify the maximum acceptable dose to fully harness these compounds in drug discovery and development.

## Data Availability

No data was used for the research described in the article.

## Abbreviations

A549: Human lung carcinoma
C6a = C7: Double bond at carbon 6a and 7
CDK2/A: Cyclin-dependent kinase 2 complexed with cyclin A
CDK9/T1: Cyclin-dependent kinase 9 complexed with cyclin T1
CDKs: Cyclin-dependent kinases
CK1, CK12: Casein kinase 1 and 12
Colo205: Human colon adenocarcinoma
DYRK1A: Dual-specificity tyrosine-phosphorylation-regulated kinase 1A
ED_50_: Median effective dose (dose required to achieve 50% of the maximum effect)
HCT-116: Human colorectal carcinoma
HEL: Human erythroleukemia
HeLa: Human cervical cancer cells
HL-60: Human leukemia cells
HT-29: Human colon adenocarcinoma
HPLC: High-performance liquid chromatography
IC_50_: Half maximal inhibitory concentration
IGF-1R: Insulin-like growth factor 1 receptor
IR: Insulin receptor
KB: Human oral epidermoid carcinoma
KB-V1: Vinblastine-resistant KB cells
L-DOPA: L-dihydroxyphenylalanine
LNCaP: Human prostate carcinoma
Lu1: Human lung cancer cells
MCF-7: Human breast adenocarcinoma
MDA-231: Human triple-negative breast cancer cells
MDR: Multidrug resistance
MG-63: Human osteosarcoma cells
MOLT-4: Human T-cell leukemia
MS: Mass spectrometry
MS–MS: Tandem mass spectrometry
NCI-H187: Human small-cell lung cancer
NMR: Nuclear magnetic resonance
UV: Ultraviolet
P-388: Murine leukemia cells
PC-3: Human prostate cancer cells
PKB-β: Protein kinase B beta
PLP: Pyridoxal 5’phosphate
QG-56: Human lung squamous carcinoma
QG-90: Human lung adenocarcinoma
RERF: Human lung adenocarcinoma cells
SAM: *S-*Adenosyl methionine
SGC7901: Human gastric cancer cells
SKOV3: Human ovarian cancer cells
SW480: Human colon adenocarcinoma
syn: Synonym
T47D: Human breast cancer cells
U2OS: Human osteosarcoma cells
VEGFR1: Vascular endothelial growth factor receptor 1
